# Supplementary material for: Loss of cytoplasmic actin filaments raises nuclear actin levels to drive INO80C-dependent chromosome fragmentation
Source: Nat Commun. 2024 Nov 15;15:9910. doi: 10.1038/s41467-024-54141-0 (PMC11568269; doi:10.1038/s41467-024-54141-0)
Supplement: Supplementary file 1 — Supplementary Information [file 41467_2024_54141_MOESM1_ESM.pdf]

Hurst et al., Supplementary data

In this pdf:
Supplementary Table 1: List of factors with altered phosphorylation upon YPK inhibition +/- Zeocin
Supplementary Table 2: *S. cerevisiae* **strains used in this study**
Supplementary Table 3: Plasmids used in this study
Supplementary Figures 1 – 6

As supplementary files (Excel sheets)
Supplementary Data 1: Excel sheet of YCS assay CHEF gel quantitation
Supplementary Data 2: Excel sheet of all phosphoproteomic data from Fig. 3

Reference numbers refer to references in the main paper.

Supplementary Table 1: Ypk+/Zeocin phosphorylated proteins with roles in cytoskeleton organization
Below is a list of Ypk+ Zeocin phospho-altered proteins that function in cytoskeleton organization and whose phosphorylation status is altered by TORC2 inhibition according to ref 37 (Rispal et al., 2015).

Factors that function in cytoskeleton organization identified in our analysis	Abp1, Akl1, Ask1, Bim1, Bni5, Bud14, Bud4, Bud6, Cdc14, Cdc37, Cin8, Cnm67, Crn1, Efb1, Ent1, Hof1, Gcs1, Gic2, Gin4, Jnm1, Kcc4, Kel1, Kip2, Las17, Nap1, Pan1, Pkc1, Rbl2, Rga1, Rga2, Shp1, Shs1, Sla1, Slg1, Slm1, Spa2, Spc42, Ssk1, Stu2, Ysc84
Phospho-proteins altered by inhibition of TORC2 (ref. 37) in common with our analysis (nuclear and DNA factors in italics; cytoskeleton regulators underlined)	<u>Acf4</u>, <u>Akl1</u>, ATG20, *Bdf1,* Boi1, Boi2, <u>Bud14</u>, Ccc1, <u>Cdc14</u>, *Cyc8*, Ded1, Dnf2, *Dot6*, Efr3, <u>Ent1</u>, Flc3, <u>Gin4,</u> Gip3, Hrk1, Imh1, Ist2, Ksp1, Lcb3, Lre1, Mam3, Mds3, Mrh1, Msc3, *Msn4*, Mss4, <u>Nap1</u>, Ngr1, *Nup2, Nup60*, *Orc6*, <u>Pan1</u>, Prr1, Rck2, Reg1, Sec16, Sis2, <u>Sla1</u>, Skg1, Smi1, Smy2, *Spt5,* Ssd1, *Stb1*, Ste20, *Swi5,* Tsl1, *Whi5*

Supplementary Table 2: *S. cerevisiae* **strains used in this study**

Internal Reference	Strain name	Genetic background	Relevant Genotype	Reference
GA-1981	W303	W303: *MATa ade2-1 trp1-1 his3-11 his3-15 ura3-1 leu2-3 leu2-112 can1-100 RAD5*	wild-type	H.L. Klein
GA-2182	*arp8Δ*	S288C: *MATa his3 leu2 met15 ura3*	*arp8::kanMX4*	X. Shen
GA-2263	BY4733	BY4733; *MATa his3Δ200 leu2Δ0 met15Δ0 trp1Δ63 ura3Δ0*	wild-type	Ref. 82 Shen et al., 2000
GA-2264	*ino80Δ*	BY4733; *MATa his3Δ200 leu2Δ0 met15del0 trp1Δ63 ura3Δ0*	*ino80::TRP1*	Ref. 82 Shen et al., 2000
GA-2319	*arp6Δ*	BY4741: *MATa his3Δ1 leu2Δ0 met15Δ0 ura3Δ0*		Euroscarf
GA-3169	*swr1Δ*	BY4741: *MATa his3Δ1 leu2Δ0 met15Δ0 ura3Δ0*	*swr1::KanMX4*	Euroscarf

GA-3440	*snf2*Δ	BY4742: MATα *his3Δ1 leu2Δ0 met15Δ0 ura3Δ0*	*snf2::KanMX4*	Euroscarf
GA-4732	BY4741	BY4741: MATa *his3Δ1 leu2Δ0 met15Δ0 ura3Δ0*	wild-type	K. Shirahige
GA-4958	Mps3-GFP	*MATa trp1-1 his3-11,15 leu2-3,112 lys2D can1-100 bar1 mps3::natMX URA3::MPS3-GFP*	*MPS3-GFP*	This study
GA-4959	mps3ΔN-GFP	*MATa trp1-1 his3-11,15 leu2-3,112 lys2D can1-100 bar1 mps3::natMX URA3::*delta*75-150-mps3-GFP*	*URA3::*delta*75-150-mps3-GFP*	This study
GA-5731	AID strain	W303: *MATa ade2-1 trp1-1 his3-11 his3-15 ura3-1 leu2-3 leu2-112 can1-100 RAD5*	*ura3-1::ADH1p_osTIR1-2-9MYC (URA3)*	Ref. 34 Shimada et al. 2013
GA-5892	YPK1 *ypk2*Δ	TB50: *MATa leu2 trp1 his3 ura3*	*ypk1::KanMX4 ypk2::HIS3 pYPK1WT (URA3)*	Ref. 34 Shimada et al. 2013
GA-5893	*ypk1*-as *ypk2*Δ	TB50: *MATa leu2 trp1 his3 ura3*	*ypk1::KanMX4 ypk2::HIS3 pYPK1as (URA3)*	Ref. 34 Shimada et al. 2013
GA-6148	TOR2	BY 4741: *MATa his3Δ1 leu2Δ0 met15Δ0 ura3Δ0*	snq2::KanMX pdr5::KanMX pdr1::NAT pdr3::KanMX yap1::NatMX pdr2::LEU2 yrm1::MET	Ref. 34 Shimada et al. 2013
GA-6150	TOR2^{BHSres}	BY 4741: *MATa his3Δ1 leu2Δ0 met15Δ0 ura3Δ0*	*TOR2_V2126G snq2::KanMX pdr5::KanMX pdr1::NAT pdr3::KanMX yap1::NatMX pdr2::LEU2 yrm1::MET*	Ref. 34 Shimada et al. 2013
GA-6696	control	BY4741: *MATa his3Δ1 leu2Δ0 met15Δ0 ura3Δ0*	ydl227c (HO)::KanMX4	OpenBiosystems
GA-6716	*swi3*Δ	BY4741: *MATa his3Δ1 leu2Δ0 met15Δ0 ura3Δ0*	yjl176c (swi3) ::KanMX4	OpenBiosystems
GA-6764	Pan1-GFP	BY4741: *MATa his3Δ1 leu2Δ0 met15Δ0 ura3Δ0*	PAN1-GFP_HIS3MX6	Invitrogen
GA-6804	Las17-GFP	BY4741: *MATa his3Δ1 leu2Δ0 met15Δ0 ura3Δ0*	LAS17(YOR181W)-GFP_HIS3MX6	Invitrogen
GA-6808	*abp1Δ*	GA-4732 with *apb1::KanMX4*	*abp1Δ*	This study
GA-6809	*sla1*Δ	BY4741: *MATa his3Δ1 leu2Δ0 met15Δ0 ura3Δ0*	ybl007c (sla1) ::KanMX4	OpenBiosystems
GA-6810	PAN1-AID	GA-5731 W303: *MATa ade2-1 trp1-1 his3-11 his3-15 ura3-1 leu2-3 leu2-112 can1-100 RAD5*	*pan1::PAN1-AID_ KanMX ura3-1:: ADH1p _osTIR1-2-9MYC (URA3)*	This study
GA-6839	LAS17-AID	GA-5731 W303: *MATa ade2-1 trp1-1 his3-11 his3-15 ura3-1 leu2-3 leu2-112 can1-100 RAD5*	*las17::LAS17-AID_KanMX ura3-*	This study

			1::ADH1p_osTIR1-2-9MYC (URA3)	
GA-6897	*bni1Δ*	GA-4732 (BY4741) with *bni1::KanMX4*	*bni1Δ*	This study
GA-7631	*atp3Δ*	GA-1981 with *atp3::natMX4, RAD52-YFP, NUP49-GFP, ADE2::TetR-mCherry, lys5::LacI-CFP-TRP1, leu2::LoxP, ZWF1:cutsite (Lmn::lys5::IsceIcs::LEU2::LacO array::Lmn),*	*atp3Δ*	This study
GA-8590	*act1-129* + pACT	MATα *his3-1 leu2Δ0 ura3Δ0 MET15 LYS2*	*act1-129:HIS3MX6 + pKFW29 (CEN, URA3, ACT1)*	Ref. 78 Haarer et al., 2007
GA-8592	*act1-111* +pACT	MATα *his3-1 leu2Δ0 ura3Δ0 MET15 LYS2*	*act1-111:HIS3MX6 + pKFW29 (CEN, URA3, ACT1)*	Ref. 78 Haarer et al., 2007
GA-9045	SPC29-Ruby2	*MATα hml::ADE1 hmr::ADE1 ade3::GALHO ade1 leu2-3, 112 lys5 trp1::hisG ura3-52, LEU2::GFP-LacI MAT::lacO repeats_TRP1 SPC29-Ruby2_KanMX CFP-NUP49*		This study
GA-9247	Wild-type	BY4733: *MATa his3Δ200 leu2Δ0 met15Δ0 trp1Δ63 ura3Δ0*	*ino80::Ino80-2xFlag; ddc2::Ddc2-HA HIS3MX6; rtf1:: Rtf1-9xPK KANMX4*	ATCC-200895
GA-9250	*arp8Δ*	BY4733: *MATa his3Δ200 leu2Δ0 met15Δ0 trp1Δ63 ura3Δ0*	*ino80::Ino80-2xFlag; ddc2::Ddc2-HA HIS3MX6; rtf1:: Rtf1-9xPK KANMX4; arp8::URA3*	This study
GA-9848	*arp8Δ*	W303: *MATa ade2-1 trp1-1 his3-11 his3-15 ura3-1 leu2-3 leu2-112 CAN1*	*lys2::KanMX; arg4::KanMX; arp8::URA*	This study
GA-9875	W303	W303: *MATa ade2-1 trp1-1 his3-11 his3-15 ura3-1 leu2-3 leu2-112 can1-100 RAD5*	wild-type	Ref. 84, Ocampo et al., 2016
GA-9878	*isw1Δ isw2Δ*	W303: *MATa ade2-1 trp1-1 his3-11 his3-15 ura3-1 leu2-3 leu2-112 can1-100 RAD5*	*isw1::ADE2 isw2::LEU2*	Ref. 84, Ocampo et al., 2016
GA-9879	*chd1Δ*	W303: *MATa ade2-1 trp1-1 his3-11 his3-15 ura3-1 leu2-3 leu2-112 can1-100 RAD5*	*chd1::HIS5*	Ref. 84, Ocampo et al., 2016
GA-9882	*isw1Δ isw2Δ chd1Δ*	W303: *MATa ade2-1 trp1-1 his3-11 his3-15 ura3-1 leu2-3 leu2-112 can1-100 RAD5*	*isw1::ADE2 isw2::LEU2 chd1::HIS5*	Ref. 84, Ocampo et al., 2016
GA-9935	*act1-111* +pACT Las17-GFP	MATα *his3-1 leu2Δ0 ura3Δ0 MET15 LYS2*	*act1-111:HIS3 + pKFW29 (CEN, URA3, ACT1) Las17::Las17-GFP_KANMX6*	This study

GA-9953	*bdf1Δ*	BY4741; *MATa his3Δ1 leu2Δ0 met15Δ0 ura3Δ0* with *bdf1::KanMX4*	*bdf1Δ*	OpenBiosystems
GA-9955	*eaf1Δ*	BY4741; *MATa his3Δ1 leu2Δ0 met15Δ0 ura3Δ0*eaf*1::KanMX4*	*eaf1Δ*	OpenBiosystems
GA-9956	*eaf7Δ*	BY4741 ; *MATa his3Δ1 leu2Δ0 met15Δ0 ura3Δ00 with eaf7::KanMX4*	*eaf7Δ*	OpenBiosystems
GA-10059	*las17* (aa1-295)	GA-4732 with *las17 1-295_HIS3MX6*	*las17 (aa*1-295)	This study
GA-10636	Las17-mini degron (W303)	GA-1981 with *LAS17-3xmini-degron_KanMX URA3::ADH1p-OsTIR1*(codon optimized*)*	Las17-mini degron (W303)	This study
GA-10701	*cap2Δ*	GA-4732 with *cap2::natMX4*	*cap2Δ*	This study
GA-10709	*las17Δ cap2Δ*	GA-4732 with *las17::KanMX4 cap2::natMX4*	*las17Δ cap2Δ*	This study
GA-10710	*las17Δ cap2Δ*	GA-4732 with *las17::KanMX4 cap2::natMX4*	*las17Δ cap2Δ*	This study
GA-10712	*mps2ΔN #1*	GA-1981 with *mps2ΔN (*1-98*)*	*mps2ΔN #1*	This study
GA-10713	*mps2ΔN #2*	GA-1981 with *mps2ΔN* (1-98)	*mps2ΔN #2*	This study
GA-10903	Las17-degron	GA-9045 with *Las17-3xmini-degron_natMX URA3::ADHp1_OsTIR1*	Las17-degron	This study
GA-10905	*las17Δ cap2Δ*	strain resulting from the cross of GA-10710 and GA-4732 with *las17::KanM4 cap2::natMX4*	*las17Δ cap2Δ*	This study

Supplementary Table 3: Plasmids used in this study

Internal Ref Number	Plasmid name	Backbone	Encoded Protein	Reference
878	pCM190	2μ, URA3	---	Ref 35 Shimada et al. 2024
2957	pCM190-act1 or pACT1	pCM190	Act1	This study
	p415 GAL1	p415 Gal1	---	Ref 96 Mumberg et al, 1994
4046	p415 GAL1- Dam	p415 Gal1	Dam	This study
4047	p415-GAL1-Las17_Dam	p415 Gal1	Las17-Dam	This study
4048	p415GAL1-ORC2_dam	p415 Gal1	Orc2-Dam	This study

Supplementary Figures and Legends 1-6

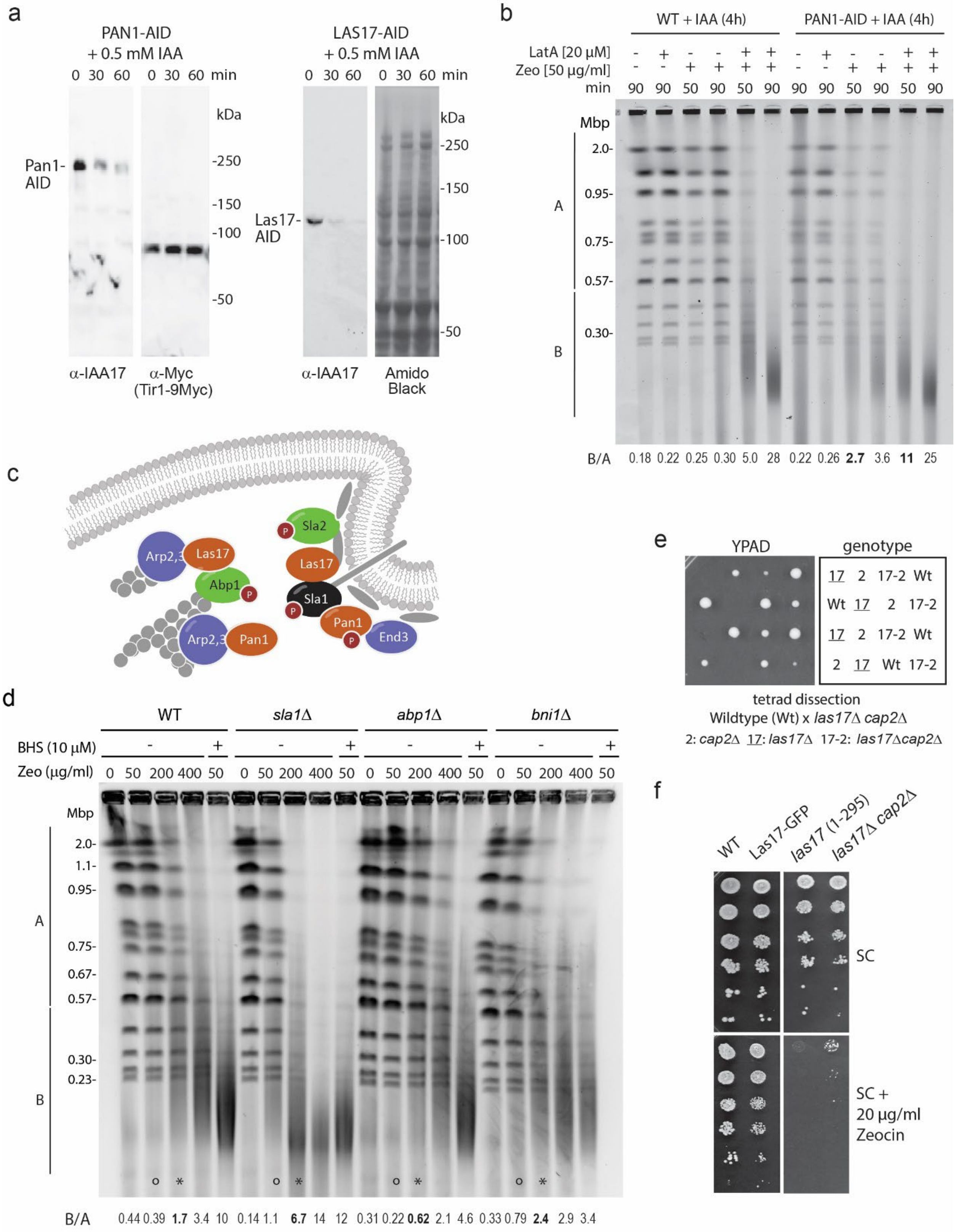

Hurst et al., Supplementary Figure 1

Supplementary Fig. 1 : Depletion of Pan1, but not Sla1, Abp1 and Bni1, sensitizes for YCS (for Fig. 4)

a) Auxin (IAA) induced degradation of Pan1-AID and Las17-AID. Conditions are in Fig. 4a,b. Total protein extracts were blotted and probed with anti-IAA17 (see Methods). Loading controls are constitutively expressed Tir1-9Myc detected by anti-Myc (9E10, Cat #MA1-980-A647, Invitrogen) and amido black staining. Full blots are shown.

b) Isogenic WT (GA-5731) and *PAN1-AID* (GA-6810) strains exponentially cultured and treated for 4h with 0.5 mM IAA to trigger Pan1-AID degradation (see a), then treated with LatA (20 μM), Zeocin (50 μg/ml), or both with IAA for indicated times. YCS was monitored and quantified as in Fig. 1b. Pan1 depletion is largely additive with LatA.

c) Scheme of the YCS-relevant phosphotargets from phosphoproteomics analysis (Fig. 3), that are involved in actin-regulated endocytosis (image based on Smythe et al. 2003; ref 64). Colors and proteins as in Fig. 3d,e.

d) Loss of regulatory factors Sla1, Abp1 and the yeast formin Bni1 does not sensitize yeast cells to Zeocin, nor prevent YCS. Exponentially cultured strains WT (GA-6696), *sla1*Δ (GA-6809), *abp1*Δ (GA-6808), and *bni1*Δ (GA-6897) were treated with indicated levels of Zeocin, or 50 μg/ml Zeocin with 10 μM BHS for 1.5h. CHEF analysis as in Fig. 1a. (o) lanes with 50 μg/ml Zeocin where Las17-degradation triggers YCS. (*) high 200μg/ml Zeocin affects *sla1*Δ more than WT. B/A ratios quantified as in Fig. 1a,b, results in Supplemental Data 1. Experiment repeated thrice with the same results.

e) Tetrad dissection of las17*Δ* cap2*Δ* double mutant. Haploid strains GA-4732 (wild-type) and GA-10710 (*las17Δ cap2Δ*) were mated, sporulated and subjected to tetrad analysis. Spores from 4 tetrads (of 20 dissections) shown after 3 days at 30°C, with relevant genotypes indicated. *las17Δ* spores were not viable, thus *cap2Δ* is a suppressor.

f) Drop assay for Zeocin sensitivity of indicated *las17* strains (left to right: GA-4732, 6804, 10059 and 10709, or WT *LAS17,* Las17-GFP, Las17 truncated at aa 295 removing polyproline domains (Urbanek et al., 2013, ref 39), and *las17*Δ *cap2*Δ, panel e). Exponentially growing cells were spotted on SC plates ± 20 μg/ml Zeocin in 5-fold serial dilutions and imaged after 3 days at 25°C; done in triplicate. Th C-terminal GFP tag on Las17 does not alter Zeocin sensitivity, while truncation does.

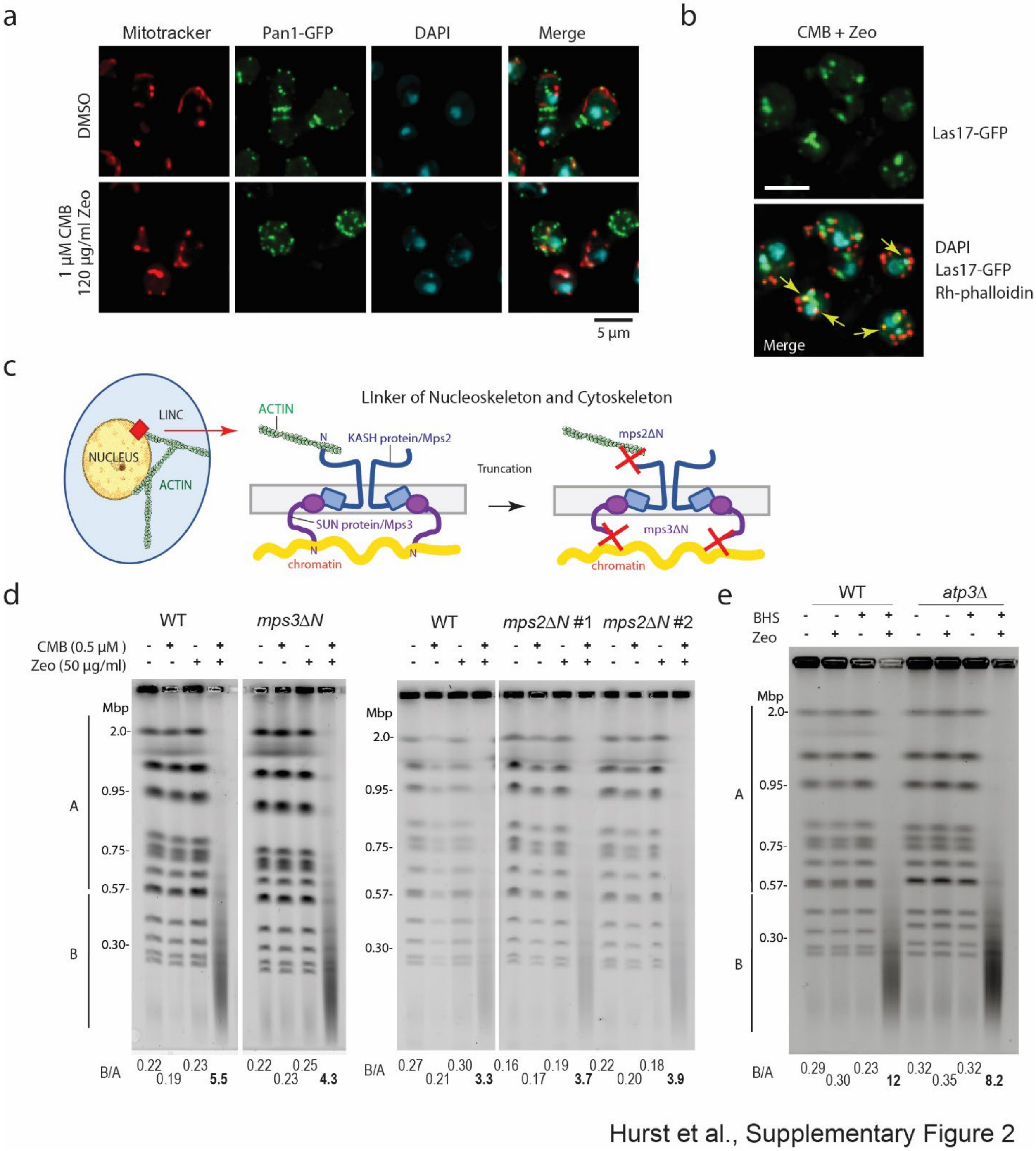

Supplementary Fig. 2: Pan1-GFP in response to YCS conditions and effects of LINC and mitochondrial mutants (Relevant to Fig. 4)

a) Pan1 mislocalizes from the bud site on BHS or LatA but does not colocalize with mitochondria. Exponentially growing cells expressing Pan1-GFP (GA-4732) were treated with 20 nM Mitotracker Red CMXRos (Thermo Scientific) for 2 h. The culture was split and treated for 90

min with DMSO or with 1 µM CMB and 150 µg/ml Zeocin to trigger YCS. Little colocalization of Pan1-GFP (green) and Mitotracker (red) was observed (would be yellow). Bar = 5µm

b) Enlarged panels from Fig. 5a, showing the relocation of Las17 under YCS conditions to nuclear-adjacent foci of unknown function (yellow arrows), potentially LINC-containing Spindle pole bodies. Bar = 5µm

c) Scheme of a yeast cell with nucleus and actin cytoskeleton connected by LINC. In yeast the KASH domain protein Mps2 connects the actin cytoskeleton to the membrane-spanning SUN domain protein Mps3, which binds both Mps2 and chromatin. Truncations investigated in panel d are marked with red X.

d) Exponentially growing cells with appropriate truncations blocking LINC complex function (*mps3ΔN*, GA-4959; and *mps2ΔN*, GA-10712/10713) were treated with Zeocin, CMB or both. Genomic DNA was analysed by CHEF gel as in Fig. 1b. The *mps2ΔN* ablates a predicted actin filament binding domain at aa 83-98 in Mps2 (Chen et al., 2029, ref 31), and the N-terminal deletion in Mps3 blocks chromatin binding (*mps3Δ75-150* (Bupp et al., 2007, ref 32). Neither mimicked TORC2 inhibition, nor showed resistance to YCS. No effect on YCS was found for a *mps2-1* ts allele at 37°C, suggesting that Las17 protects from YCS independent of the LINC complex.

e) An isogenic parental strain (GA-1981) and mutant *atp3*Δ (GA-7631), ablated for the F1F0 ATPase and oxidative phosphorylation, were grown on glucose-containing media and incubated 90 min with Zeocin (60µg/ml) and BHS (10 µM). Genomic DNA was subjected to CHEF gel analysis, and fragmentation was quantified as in Fig. 1b. The mutant and isogenic parent strain were identical. Complete loss of the mitochondrial genome (rho° strain) underwent YCS, suggesting that Las17's impact on the mitochondrial genome is irrelevant. CHEF gel quantitation is in Supplementary Data 1.

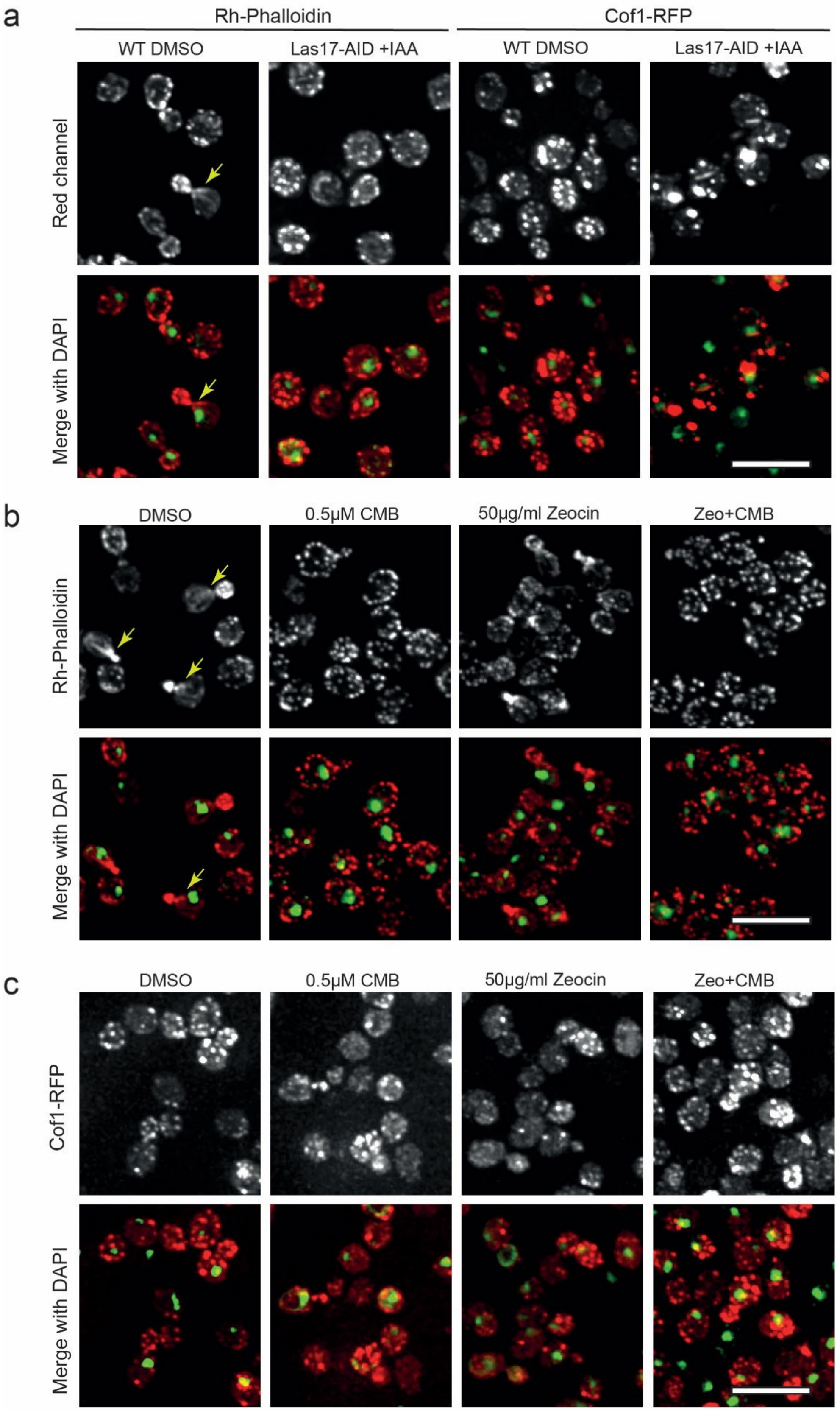

Hurst et al., Supplementary Figure 3

Supplementary Fig. 3 Imaging of cofilin-RFP and rhodamine-phalloidin in control and YCS inducing conditions (Relevant for Figs. 5,6)

a) At left, exponentially growing cells (GA-5731 (AID control) and GA-6840 (Las17-AID), were treated as indicated (± 0.25 mM IAA for Las17-AID), fixed with 4% PFA and stained with Rh-phalloidin (red) and DAPI (green). At right the same strains carrying Cof1-RFP plasmid were processed similarly, skipping the Phalloidin stain. Images were acquired and the budding index as well as the number of cells with filaments were determined manually by 3 independent operators in a blinded fashion. Bar = 10 µm
b) Exponentially growing GA-1981 (WT) cells were treated as indicated 50 µg/ml Zeocin ± 0.5 µM CMB for 90 min and were fixed with 4% PFA and stained with Rh-phalloidin (red) and DAPI (green). Images were acquired and the budding index as well as the number of cells with filaments were determined manually by 3 independent operators. Bar= 10 µm
c) Exponentially growing GA-1981 (WT) carrying Cof1-RFP plasmid were treated as indicated 50 µg/ml Zeocin ± 0.5 µM CMB for 90 min and were fixed with 4% PFA and visualized for Cof1-RFP (red) and DAPI (green). Bar = 10 µm

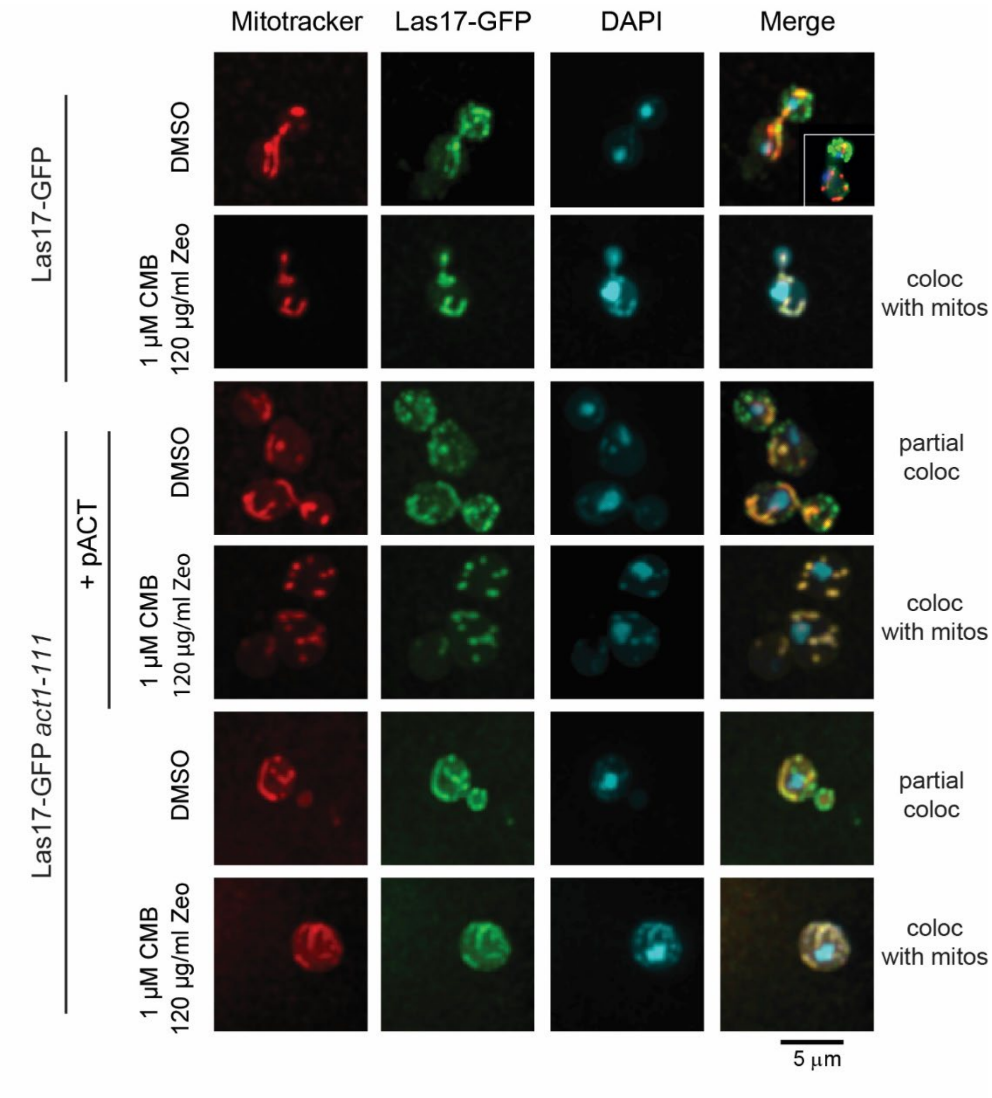

Supplementary Fig. 4 Las17 localization responds to TORC2 inhibition in *act1-111* (relevant to Fig. 7)

Imaging of Las17-GFP in the *act-111* mutant (GA-9935). Cells were treated as indicated, stained with Mitotracker, fixed and then stained with DAPI. The inset in the DMSO control shows another cell in which Las17-GFP is largely distinct from mitochondria (red and green partially distinct), while under YCS conditions Las17-GFP colocalizes strongly with mitochondria (yellow signal). The untreated *act1-111* mutant shows partial Las17-GFP colocalization with mitochondria, but the overlap is still stronger under YCS conditions. The nuclei show no enrichment for Las17-GFP (blue-green overlap). Bar = 5 μM.

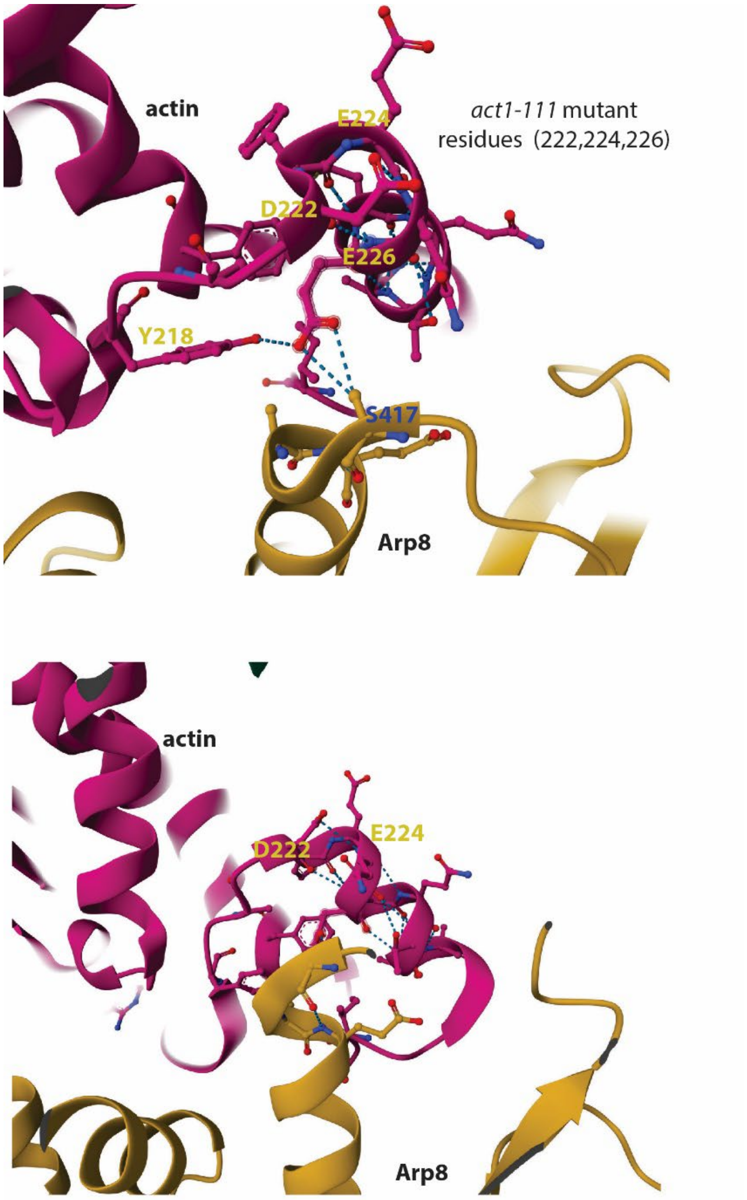

Hurst et al., Supplemental Figure 5

Supplemental Fig. 5 The *act1-111* mutations could affect Arp8 interaction (relevant to Fig. 7)
Shown is an enlarged view at atomic resolution of the interaction of actin (magenta) and Arp8 (gold) subunits in the INO80C complex, in which we have labeled the three residues mutated in the *act1-111* mutant (D222, E224, E226) and the relevant tyrosine Y218 that forms a bridge to Arp8 residue S417. The interface is rotated in the lower panel. See relevant structure references (Kapoor et al., 2013, ref 80; Kapoor et al., 2014, ref 13; and Zhang et al., 2019, ref 81).

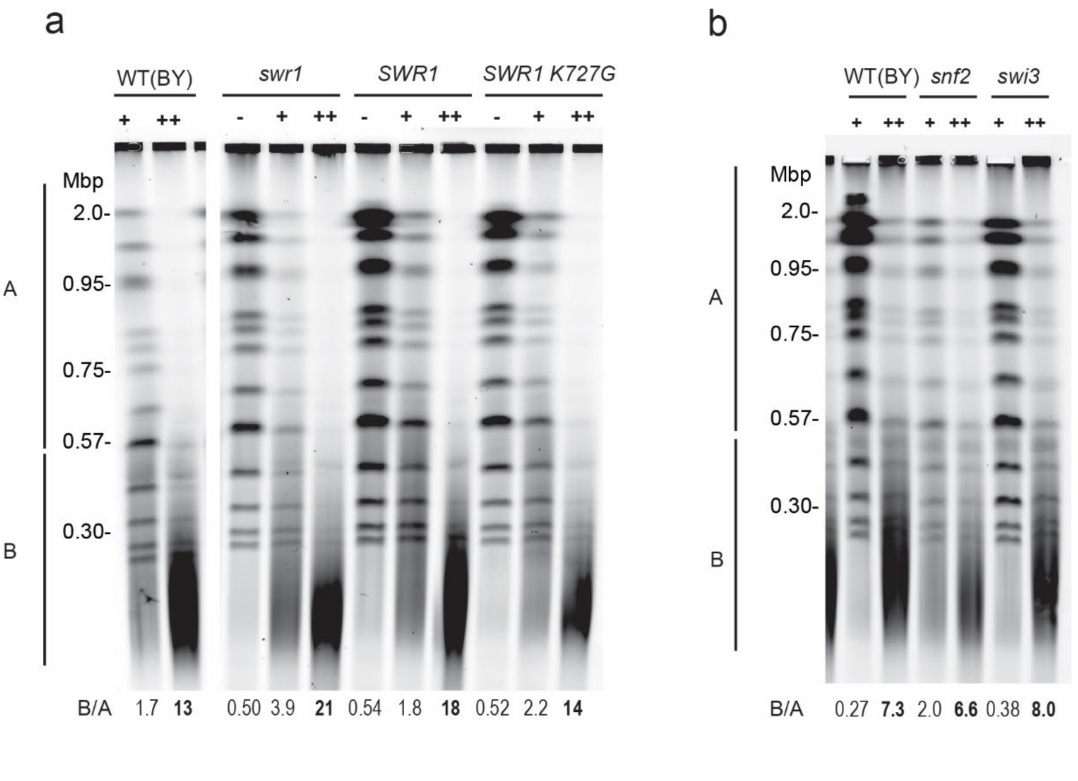

Supplementary Fig. 6 SNF2 and SWR-C mutations do not alter YCS (relevant to Fig. 8)

a) The deletion of the SWR-C catalytic subunit *swr1*Δ in the BY background (WT= GA-4732 and *swr1*Δ = GA-3169) as well as its complementation with *SWR1* or the ATPase deficient *SWR1* K727G allele on Cen plasmids were exposed to either mild (+ = 0.5 µM CMB, 60 µg/ml Zeocin) or strong (++ = 1 µM CMB, 120 µg/ml Zeocin) shattering conditions for 90 min prior to processing for CHEF gel analysis. Quantitation is performed as in Fig. 1b and values are presented in Supplementary Data 1. Neither allele impairs efficient YCS, and *swr1*Δ accentuates fragmentation, as shown by bold B/A values (B/A = 21 vs 13, 18 or 14). Experiments were repeated 2 times.

b) Deletions of remodeling complex subunits Snf2 (*snf2*Δ, GA-3440) and Swi3 (*swi3*Δ, GA-6716) of the SWI/SNF chromatin remodeling complex in the BY background (GA-2263) were subjected to mild (+ = 0.5 µM CMB, 60 µg/ml Zeocin) or strong (++ = 1 µM CMB, 120 µg/ml Zeocin) shattering conditions for 90 min. Neither of the tested deletion mutants conferred resistance, but the *snf2*Δ strain is more sensitive to lower CMB/Zeo concentrations (on 0.5 µM CMB+ 60 µg/ml Zeocin, B/A = 2.0 vs 0.27 in wild-type cells). Quantitation was performed as in Fig. 1b and values are presented in Supplementary Data 1. Experiments were repeated 3 times.

Supplementary Data 1: Quantitation of CHEF gels shown in main and supplementary figures

All CHEF gels presented in the paper are presented in their entirety and were quantified as described in Methods. The calculation of B/A values is presented in this Excel spreadsheet. The correlation of B/A ratio with estimated double-strand breaks is described in Shimada et al., ref. [35].

Supplementary Data 2 : Excel sheet of phosphoproteomic data from Fig. 3

The phosphoproteomic experiment shown in Figure 3 was performed in triplicate. The detected levels of phosphopepetides and identification of the protein they are derived from are presented in its entirety in this excel table. See Methods and text for description of the sample preparation and mass spectroscopy, which was performed using a label-free method as in refs[57,58].
