# Supplementary material for: Loss of cytoplasmic actin filaments raises nuclear actin levels to drive INO80C-dependent chromosome fragmentation
Source: Nat Commun. 2024 Nov 15;15:9910. doi: 10.1038/s41467-024-54141-0 (PMC11568269; doi:10.1038/s41467-024-54141-0)
Supplement: Supplementary file 2 — Description of Additional Supplementary Files [file 41467_2024_54141_MOESM2_ESM.pdf]

Description of Additional Supplementary Files

Supplementary Data 1: Quantitation of CHEF gels shown in main and supplementary figures

All CHEF gels presented in the paper are presented in their entirety and were quantified as described in Methods. The calculation of B/A values is presented in this Excel spreadsheet. The correlation of B/A ratio with estimated double-strand breaks is described in Shimada et al., ref. [35].

Supplementary Data 2: Excel sheet of phosphoproteomic data from Fig. 3

The phosphoproteomic experiment shown in Figure 3 was performed in triplicate. The detected levels of phosphopepetides and identification of the protein they are derived from are presented in its entirety in this excel table. See Methods and text for description of the sample preparation and mass spectroscopy, which was performed using a label-free method as in refs[57,58].
