# Supplementary material for: Loss of cytoplasmic actin filaments raises nuclear actin levels to drive INO80C-dependent chromosome fragmentation
Source: Nat Commun. 2024 Nov 15;15:9910. doi: 10.1038/s41467-024-54141-0 (PMC11568269; doi:10.1038/s41467-024-54141-0)
Supplement: Supplementary file 5 — Reporting Summary [file 41467_2024_54141_MOESM5_ESM.pdf]

nature portfolio

Corresponding author(s): Susan M Gasser

Last updated by author(s): Sep 30, 2024

Reporting Summary

Nature Portfolio wishes to improve the reproducibility of the work that we publish. This form provides structure for consistency and transparency in reporting. For further information on Nature Portfolio policies, see our Editorial Policies and the Editorial Policy Checklist.

Statistics

For all statistical analyses, confirm that the following items are present in the figure legend, table legend, main text, or Methods section.

n/a	Confirmed	
☐	☒	The exact sample size (*n*) for each experimental group/condition, given as a discrete number and unit of measurement
☐	☒	A statement on whether measurements were taken from distinct samples or whether the same sample was measured repeatedly
☐	☒	The statistical test(s) used AND whether they are one- or two-sided<br>*Only common tests should be described solely by name; describe more complex techniques in the Methods section.*
☒	☐	A description of all covariates tested
☐	☒	A description of any assumptions or corrections, such as tests of normality and adjustment for multiple comparisons
☐	☒	A full description of the statistical parameters including central tendency (e.g. means) or other basic estimates (e.g. regression coefficient) AND variation (e.g. standard deviation) or associated estimates of uncertainty (e.g. confidence intervals)
☐	☒	For null hypothesis testing, the test statistic (e.g. *F*, *t*, *r*) with confidence intervals, effect sizes, degrees of freedom and *P* value noted<br>*Give P values as exact values whenever suitable.*
☒	☐	For Bayesian analysis, information on the choice of priors and Markov chain Monte Carlo settings
☒	☐	For hierarchical and complex designs, identification of the appropriate level for tests and full reporting of outcomes
☒	☐	Estimates of effect sizes (e.g. Cohen's *d*, Pearson's *r*), indicating how they were calculated

Our web collection on statistics for biologists contains articles on many of the points above.

Software and code

Policy information about availability of computer code

Data collection	Imaging data collection is done using standard software and tools are described in Methods
Data analysis	Image analysis was performed with standard software that is described in the Methods

For manuscripts utilizing custom algorithms or software that are central to the research but not yet described in published literature, software must be made available to editors and reviewers. We strongly encourage code deposition in a community repository (e.g. GitHub). See the Nature Portfolio guidelines for submitting code & software for further information.

Data

Policy information about availability of data

All manuscripts must include a data availability statement. This statement should provide the following information, where applicable:

- Accession codes, unique identifiers, or web links for publicly available datasets
- A description of any restrictions on data availability
- For clinical datasets or third party data, please ensure that the statement adheres to our policy

all data is included in the submission either in figures or as excel files

Research involving human participants, their data, or biological material

Policy information about studies with human participants or human data. See also policy information about sex, gender (identity/presentation), and sexual orientation and race, ethnicity and racism.

Reporting on sex and gender	na
Reporting on race, ethnicity, or other socially relevant groupings	na
Population characteristics	na
Recruitment	na
Ethics oversight	na

Note that full information on the approval of the study protocol must also be provided in the manuscript.

Field-specific reporting

Please select the one below that is the best fit for your research. If you are not sure, read the appropriate sections before making your selection.

☒ Life sciences ☐ Behavioural & social sciences ☐ Ecological, evolutionary & environmental sciences

For a reference copy of the document with all sections, see nature.com/documents/nr-reporting-summary-flat.pdf

Life sciences study design

All studies must disclose on these points even when the disclosure is negative.

Sample size	for each experiment number of cells counted is included
Data exclusions	none except out-of-focus planes in images
Replication	indicated in legends and supplemental material
Randomization	not relevant
Blinding	double blind approaches were used for quantifying images, usually with three individuals

Reporting for specific materials, systems and methods

We require information from authors about some types of materials, experimental systems and methods used in many studies. Here, indicate whether each material, system or method listed is relevant to your study. If you are not sure if a list item applies to your research, read the appropriate section before selecting a response.

Materials & experimental systems

n/a	Involved in the study	
☐	☒	Antibodies
☐	☒	Eukaryotic cell lines
☒	☐	Palaeontology and archaeology
☒	☐	Animals and other organisms
☒	☐	Clinical data
☒	☐	Dual use research of concern
☒	☐	Plants

Methods

n/a	Involved in the study	
☒	☐	ChIP-seq
☒	☐	Flow cytometry
☒	☐	MRI-based neuroimaging

Antibodies

Antibodies used	aAntibodies used are either commercial (95kd nuclear pore protein by Mab414; Abcam, ab24609) used 1:1000, Rabbit polyclonal antibody against hACT1 (aa 346 – 375; My BioSource, BSS9231831) used 1:1000, rat anti-Tubulin monoclonal antibody (Abcam, ab6161, YOL 1/34) used 1:10000 dilution, anti-IAA17 (gift of M. Kanemaki, NIG, Japan), used 1:1000, or anti-H2A-S129-phospho

	antibody (rabbit polyclonal), 1 : 1000 dilution95 validated in cited reference. Also where indicated mouse anti-actin (monoclonal); (Clone C4, MAB1501, Millipore), was used at 1: 4000 dilution.
Validation	negative controls are aways used to confirm specificity, papers cited when validation was done earlier

Eukaryotic cell lines

Policy information about cell lines and Sex and Gender in Research

Cell line source(s)	Human dermal fibroblasts neonatal (HDFn) cells (Thermo Fisher Scientific) and HCT116 (ATCC, CCL-247)
Authentication	HCT116 were authenticated by sequencing, Novartis
Mycoplasma contamination	none
Commonly misidentified lines (See ICLAC register)	na

Plants

Seed stocks	na
Novel plant genotypes	na
Authentication	na
