# Supplementary material for: Loss of cytoplasmic actin filaments raises nuclear actin levels to drive INO80C-dependent chromosome fragmentation
Source: Nat Commun. 2024 Nov 15;15:9910. doi: 10.1038/s41467-024-54141-0 (PMC11568269; doi:10.1038/s41467-024-54141-0)

PKF052-766-NX-1 x Zeocin Anti-Cancer (blank) HCT116

Single Agent - Zeocin

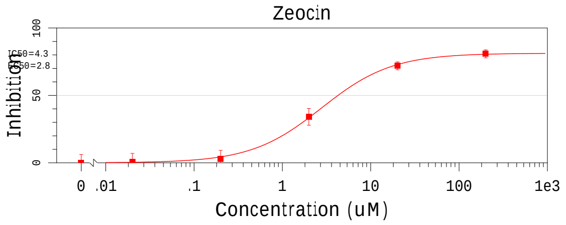

Single Agent - PKF052-766-NX-1

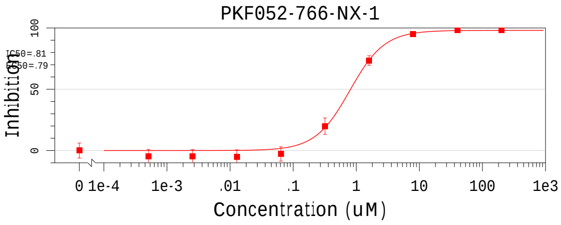

Combination - Synergy Score: 3 (.27); Best CI: .52 (.34)

Inhibition

PKF052-766-NX-1 (uM) (rows) × Zeocin (uM) (columns); N=1

PKF052-766-NX-1 (uM) \ Zeocin (uM)	0	.02	.2	2	20	200
200	98	99	99	99	99	100
	98	99	99	99	100	101
8	95	95	95	96	97	100
	73	70	74	81	82	86
.32	20	18	21	55	72	84
	-3	-4	3	52	73	83
.013	-5	-6	-4	47	75	85
	-5	-4	-3	48	74	84
5.1e-4	-5	-4	-4	52	73	84
0	0	1	3	34	72	81

ADD Excess Inhibition

Vol=.582(.26) Chi2=2.6

PKF052-766-NX-1 (uM) \ Zeocin (uM)	0	.02	.2	2	20	200
200	0	1	1	1	1	2
	0	1	1	1	2	3
8	-1	0	0	0	1	4
	0	-5	0	5	2	5
.32	1	-2	-3	9	-2	3
	-5	-6	-4	16	0	2
.013	-5	-7	-9	14	2	4
	-5	-4	-8	15	1	3
5.1e-4	-5	-5	-8	19	0	3
0	0	0	-1	1	-1	0

Inhibition Error

PKF052-766-NX-1 (uM) \ Zeocin (uM)	0	.02	.2	2	20	200
200	1	1	1	1	1	1
	1	1	1	1	1	1
8	1	2	2	1	2	1
	4	4	4	3	2	2
.32	7	7	9	5	3	2
	6	6	7	5	3	2
.013	6	6	6	5	3	2
	6	6	6	5	3	2
5.1e-4	6	6	6	5	2	2
0	6	6	6	6	3	3

Isobologram

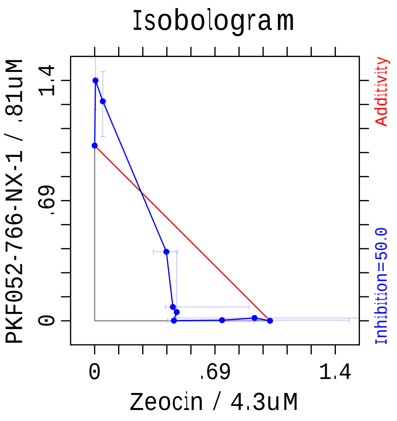

NVP-CCG168-NX-3 x Zeocin Anti-Cancer (blank) HCT116

Single Agent - Zeocin

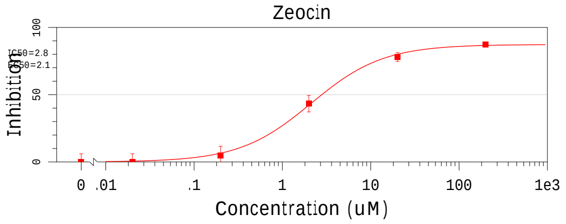

Single Agent - NVP-CCG168-NX-3

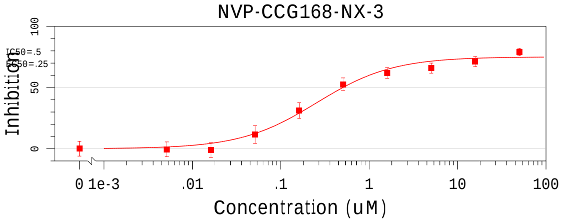

Combination - Synergy Score: 6.2 (.31); Best CI: .56 (.12)

Inhibition

N=1

NVP-CCG168-NX-3 (uM) \ Zeocin (uM)	0	.02	.2	2	20	200
50	79	78	80	88	92	100
	71	70	71	83	92	98
5.1	66	66	67	81	89	97
	62	64	64	81	90	97
.51	53	54	57	79	87	95
	31	34	42	72	84	94
.051	12	8	18	64	81	93
	-1	0	6	56	81	91
.0051	0	0	3	56	80	89
0	0	0	5	43	78	87

ADD Excess Inhibition

Vol=2.51(.29) Chi2=13

NVP-CCG168-NX-3 (uM) \ Zeocin (uM)	0	.02	.2	2	20	200
50	4	3	5	13	12	13
	-3	-4	-3	9	12	11
5.1	-6	-6	-5	9	9	10
	-3	-1	-2	14	10	10
.51	3	4	6	20	7	9
	2	5	10	22	4	8
.051	-1	-5	0	18	1	6
	-6	-4	-5	13	1	4
.0051	-2	-2	-5	13	0	2
0	0	-1	-2	1	-2	1

Inhibition Error

NVP-CCG168-NX-3 (uM) \ Zeocin (uM)	0	.02	.2	2	20	200
50	3	3	3	2	2	1
	4	4	4	3	2	1
5.1	4	4	5	3	2	2
	4	5	4	3	2	2
.51	5	5	6	3	2	2
	6	7	7	4	3	2
.051	7	8	7	5	3	2
	6	7	7	6	3	3
.0051	6	7	7	5	3	3
0	6	6	7	6	3	2

Isobologram

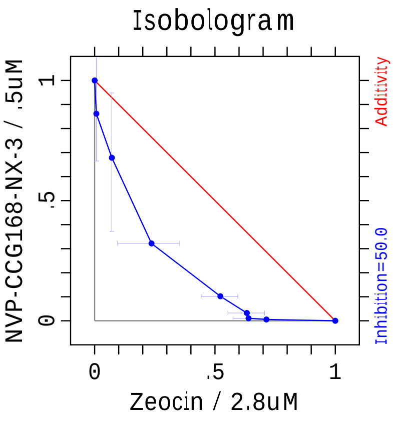

PKF090-346-NX-1 x Zeocin Anti-Cancer (blank) HCT116

Single Agent - Zeocin

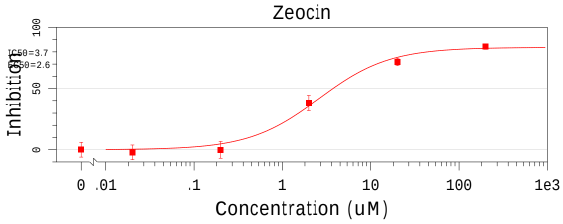

Single Agent - PKF090-346-NX-1

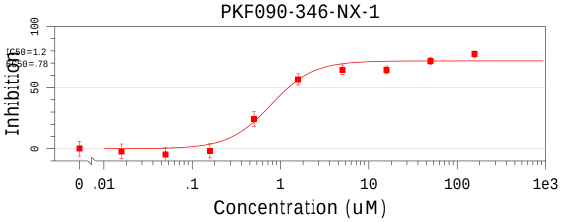

Combination - Synergy Score: 5.1 (.28); Best CI: .54 (.073)

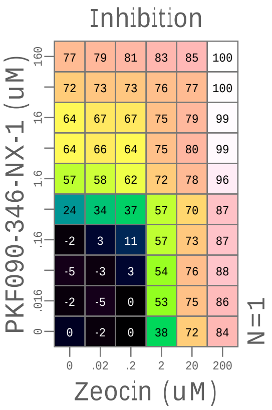

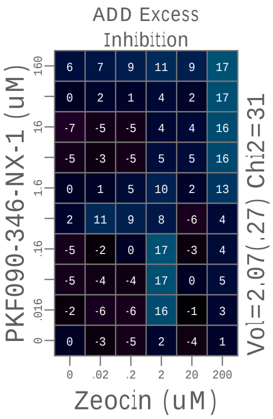

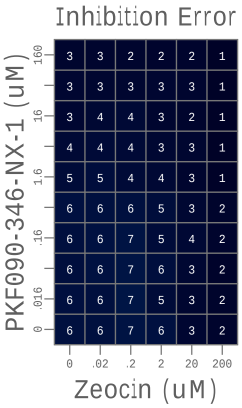

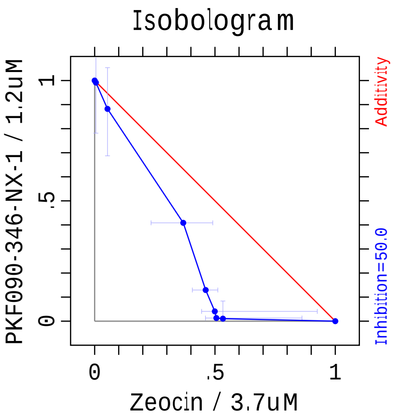

PKF222-666-NX-1 x Zeocin Anti-Cancer (blank) HCT116

Single Agent - Zeocin

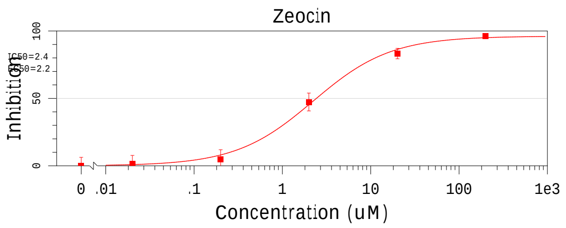

Single Agent - PKF222-666-NX-1

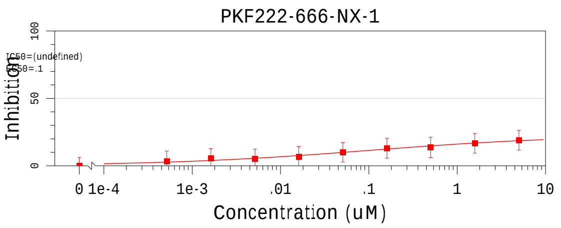

Combination - Synergy Score: 4.6 (.34); Best CI: .54 (.075)

Inhibition

N=1

PKF222-666-NX-1 (uM) \ Zeocin (uM)	0	.02	.2	2	20	200
5	19	20	24	67	85	100
	17	19	25	67	87	99
.5	14	16	20	70	88	99
	13	14	15	67	88	99
.051	10	11	6	71	87	99
	7	11	17	71	84	98
.0051	5	5	13	67	86	99
	5	3	13	66	85	98
5.2e-4	3	5	12	65	85	96
0	0	1	5	47	83	96

ADD Excess Inhibition

Vol=2.65(.38) Chi2=4.7

PKF222-666-NX-1 (uM) \ Zeocin (uM)	0	.02	.2	2	20	200
5	0	1	4	21	-2	5
	0	2	8	21	1	4
.5	-1	1	4	24	1	4
	1	1	1	21	2	4
.051	0	1	-5	24	1	4
	-1	3	6	25	-2	3
.0051	0	-1	4	21	-1	4
	2	-1	5	20	-1	3
5.2e-4	0	2	4	19	-1	1
0	0	0	-3	2	-3	1

Inhibition Error

PKF222-666-NX-1 (uM) \ Zeocin (uM)	0	.02	.2	2	20	200
5	7	7	7	5	4	2
	7	7	9	6	3	2
.5	8	8	8	5	3	2
	7	8	8	5	3	2
.051	7	8	8	5	3	2
	7	7	8	4	3	2
.0051	7	8	8	5	3	2
	7	7	8	5	3	2
5.2e-4	8	7	8	5	3	2
0	6	6	7	7	4	2

Isobologram

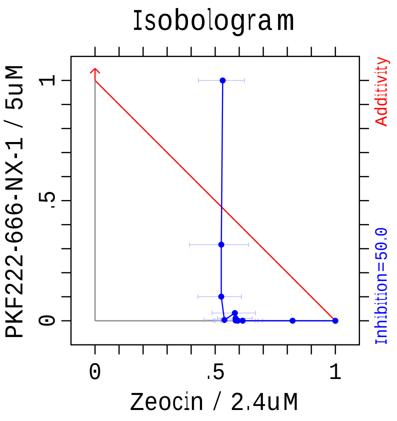

Supplement: Supplementary file 7 — Source Data [file 41467_2024_54141_MOESM7_ESM.zip › Fig 2 e_HCT116_synergy.pdf]
