# Supplementary material for: Loss of cytoplasmic actin filaments raises nuclear actin levels to drive INO80C-dependent chromosome fragmentation
Source: Nat Commun. 2024 Nov 15;15:9910. doi: 10.1038/s41467-024-54141-0 (PMC11568269; doi:10.1038/s41467-024-54141-0)
Supplement: Supplementary file 7 — Source Data [file 41467_2024_54141_MOESM7_ESM.zip › Fig 2a/Suppl data text 2a.pdf]

Fig 2a : effect of sequential treatments with Zeocin and Latrunculin B on DNA damage checkpoint activation

The experiment was performed in duplicate and the Western blots for gamma H2AX and tubulin are shown. As the results were very similar only the results to the right were shown. In the figure the intensity of signal was uniformly enhanced for the histone signal. This experiment was repeated multiple times with titrations of LatB and also LatA was tested with similar results. For another example with quantitation see panel 2c.

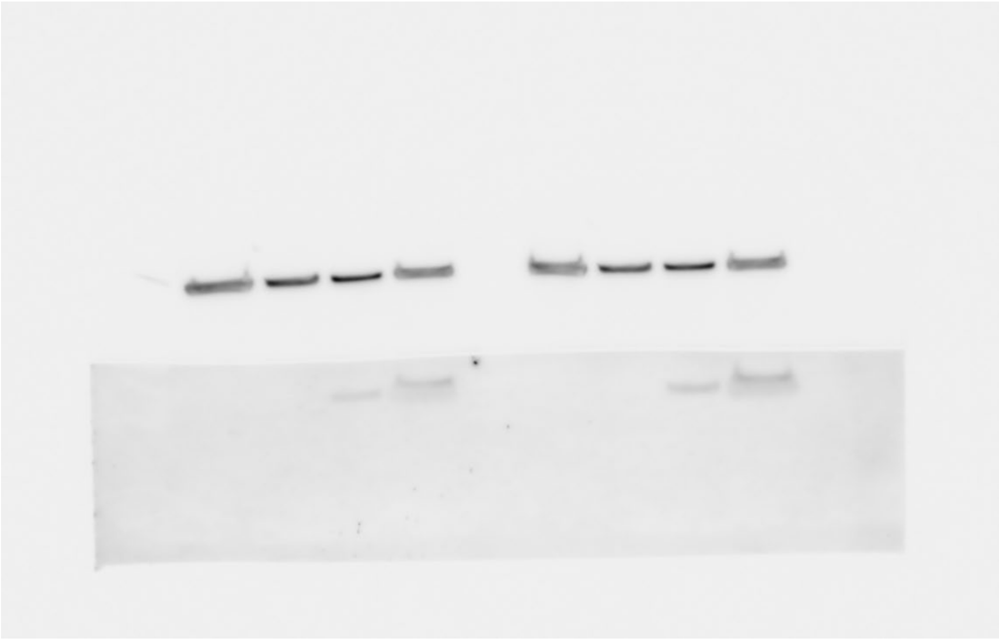

Anti-Tubulin

Anti-γH2AX

DMSO +LatB +Zeo +Z+L   DMSO +LatB +Zeo +Z+L
