# Supplementary material for: Loss of cytoplasmic actin filaments raises nuclear actin levels to drive INO80C-dependent chromosome fragmentation
Source: Nat Commun. 2024 Nov 15;15:9910. doi: 10.1038/s41467-024-54141-0 (PMC11568269; doi:10.1038/s41467-024-54141-0)
Supplement: Supplementary file 7 — Source Data [file 41467_2024_54141_MOESM7_ESM.zip › Fig 2b repeat/repeat of exp fig 2b.pdf]

Repeat of experiment shown in Figure 2b. The single drugs caused no cell death and no cell suspension at any of the concentrations tested (1-300 micrograms/ml Zeocin or up to 300nM Lat B)

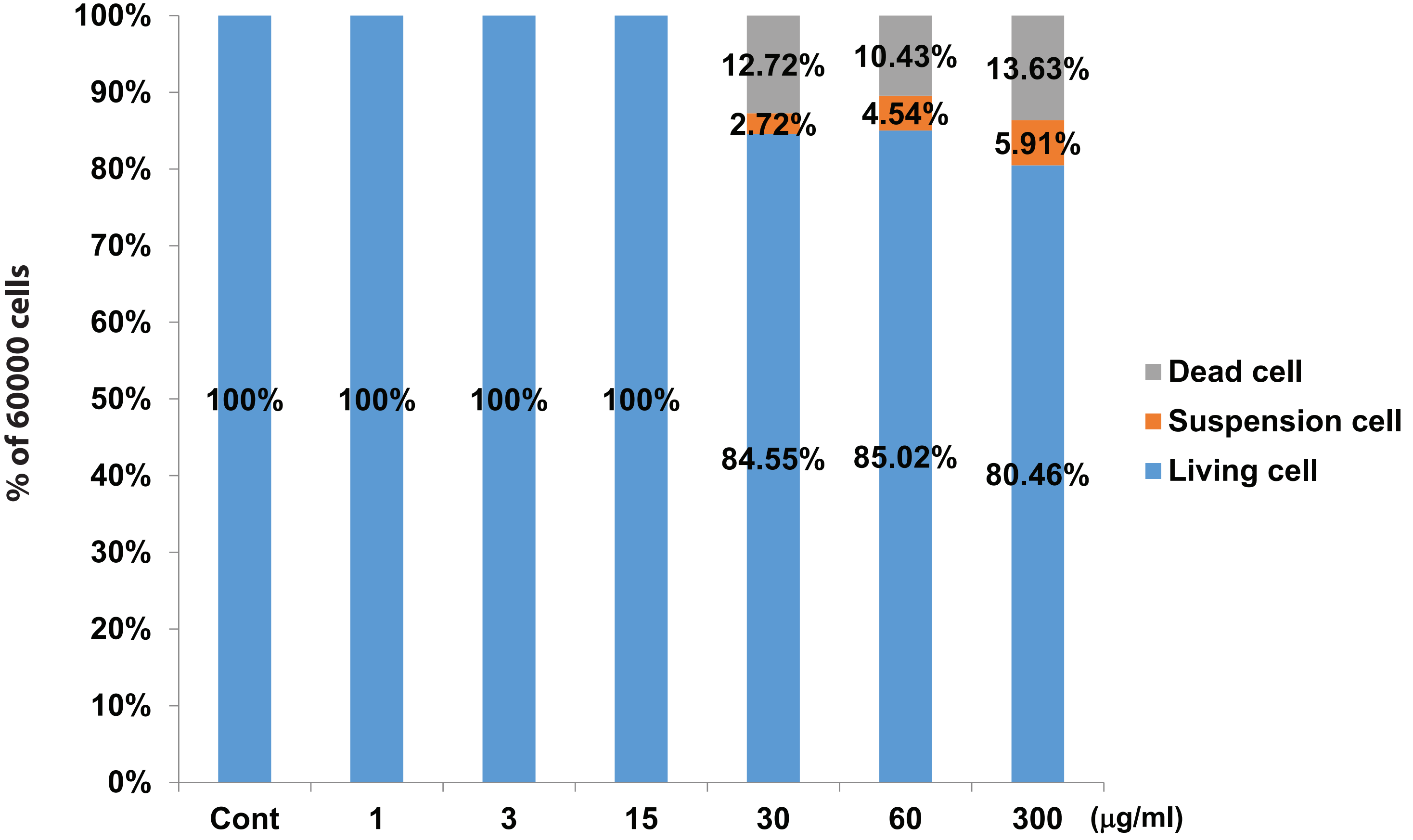

Cells incubated 1h in 300nM LatB plus a titration of Zeocin
