# Supplementary figures and images for: Loss of cytoplasmic actin filaments raises nuclear actin levels to drive INO80C-dependent chromosome fragmentation
Source: Nat Commun. 2024 Nov 15;15:9910. doi: 10.1038/s41467-024-54141-0 (PMC11568269; doi:10.1038/s41467-024-54141-0)

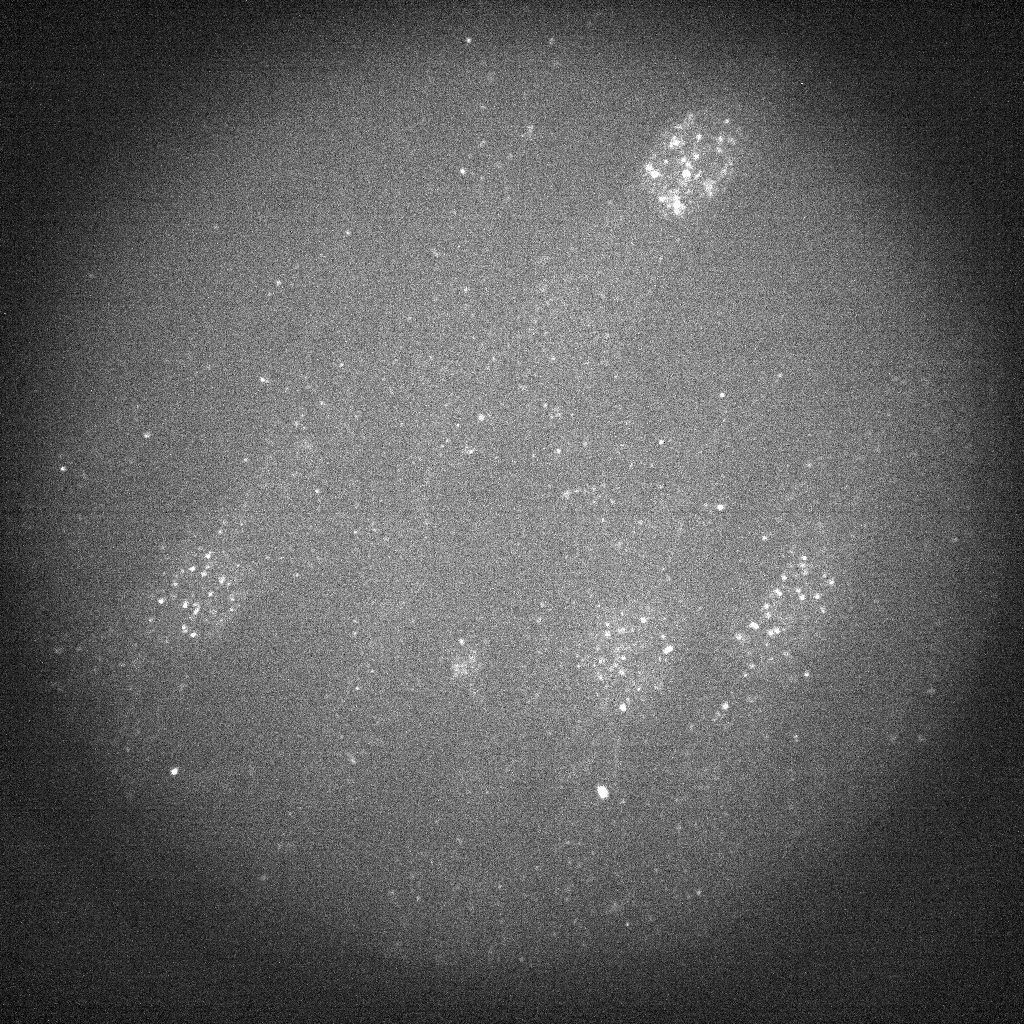

Supplement: Supplementary file 7 — Source Data [file 41467_2024_54141_MOESM7_ESM.zip › Fig 2c/control/Fig.2C control anti-phospho-Histone H2A.X.tif]

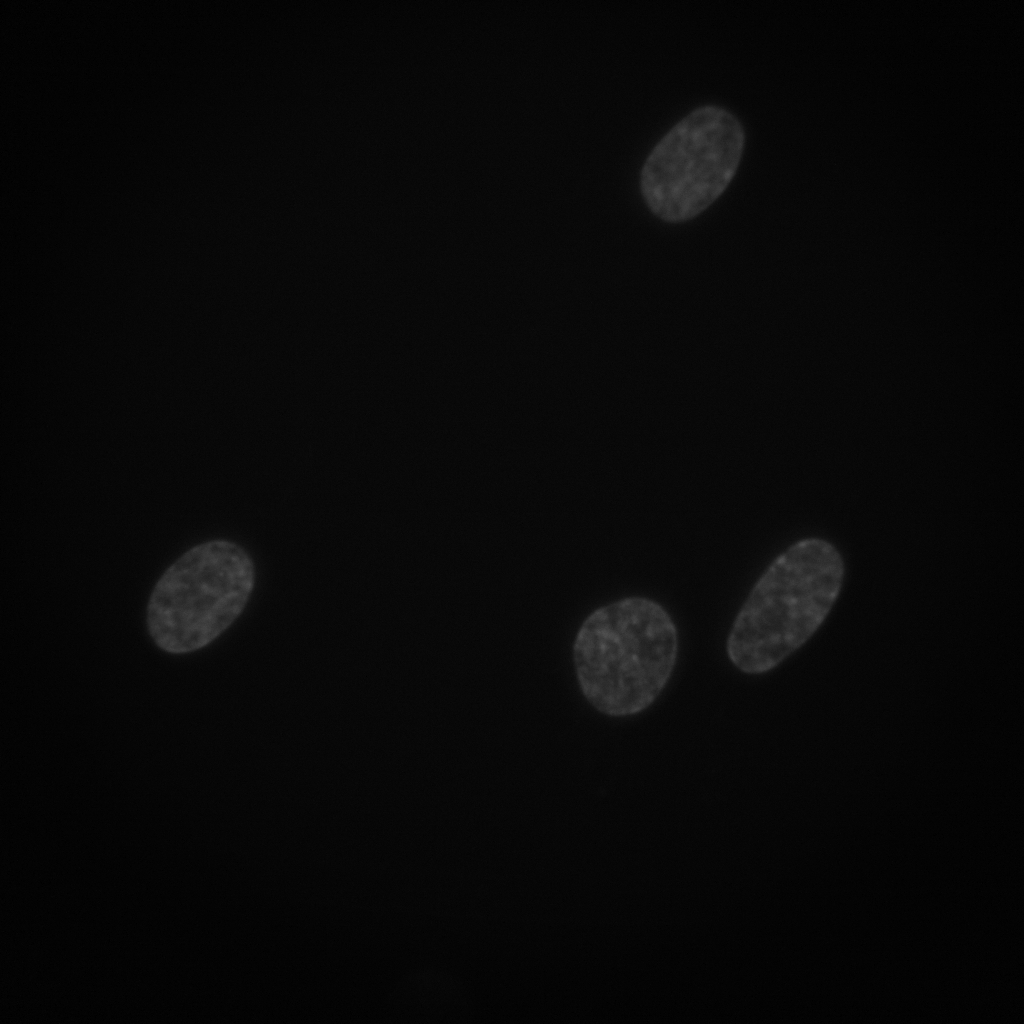

Supplement: Supplementary file 7 — Source Data [file 41467_2024_54141_MOESM7_ESM.zip › Fig 2c/control/Fig.2C control DAPI.tif]

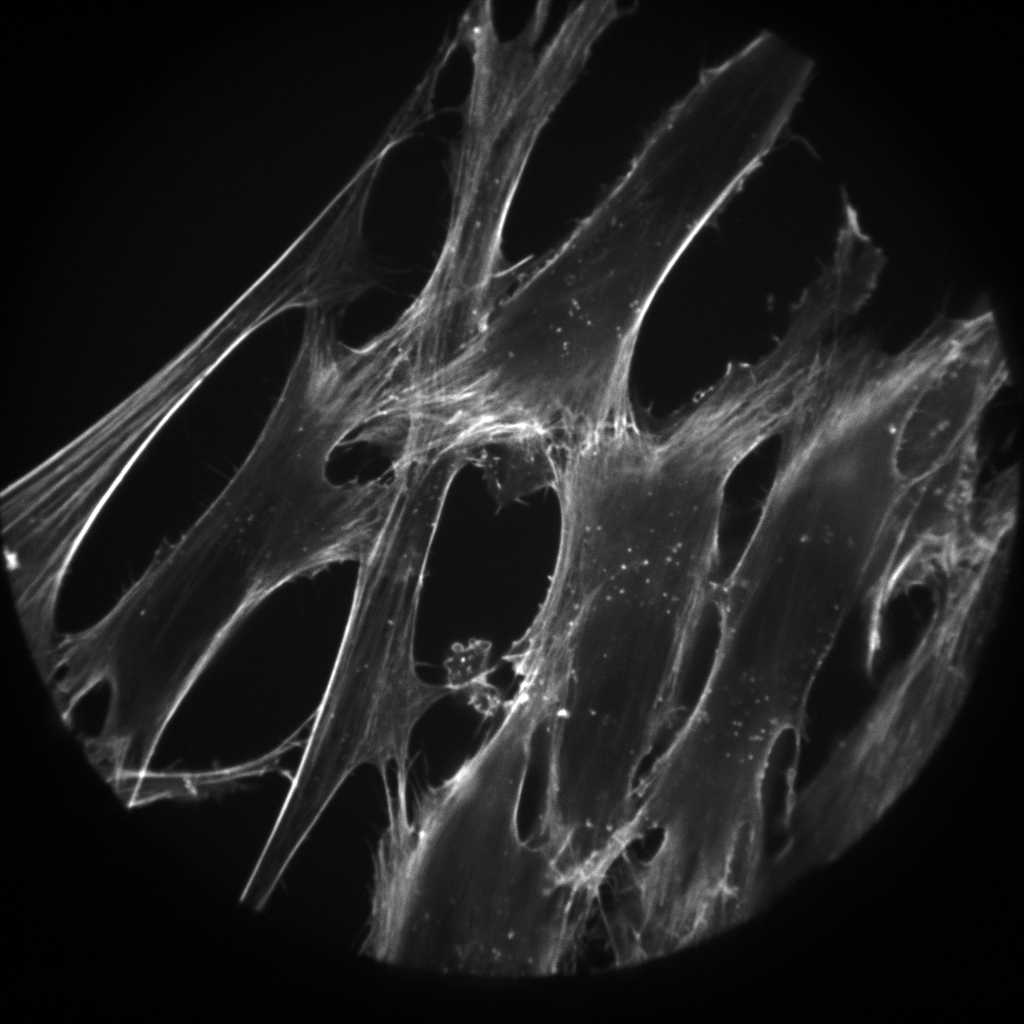

Supplement: Supplementary file 7 — Source Data [file 41467_2024_54141_MOESM7_ESM.zip › Fig 2c/control/Fig.2C control SiR-actin.tif]

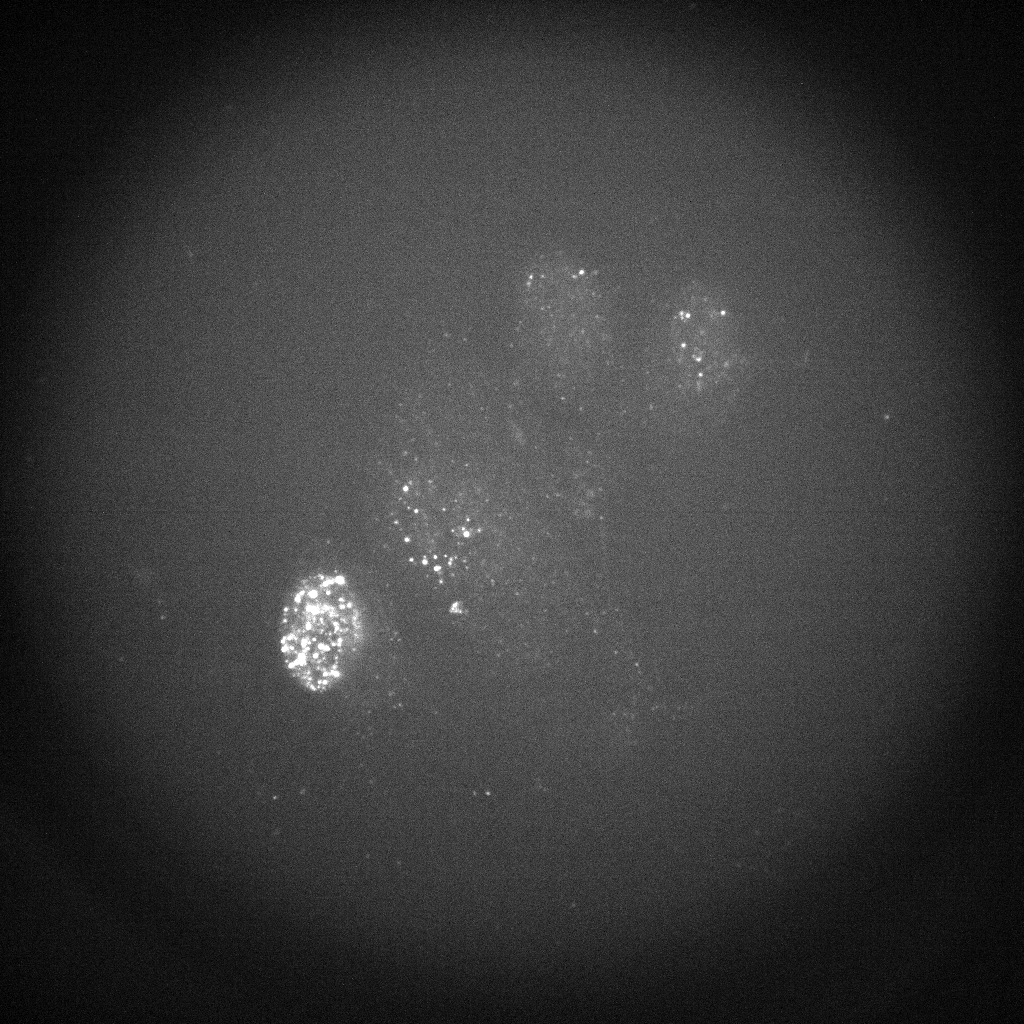

Supplement: Supplementary file 7 — Source Data [file 41467_2024_54141_MOESM7_ESM.zip › Fig 2c/LatB/Fig.2C LatB anti-phospho-Histone H2A.X.tif]

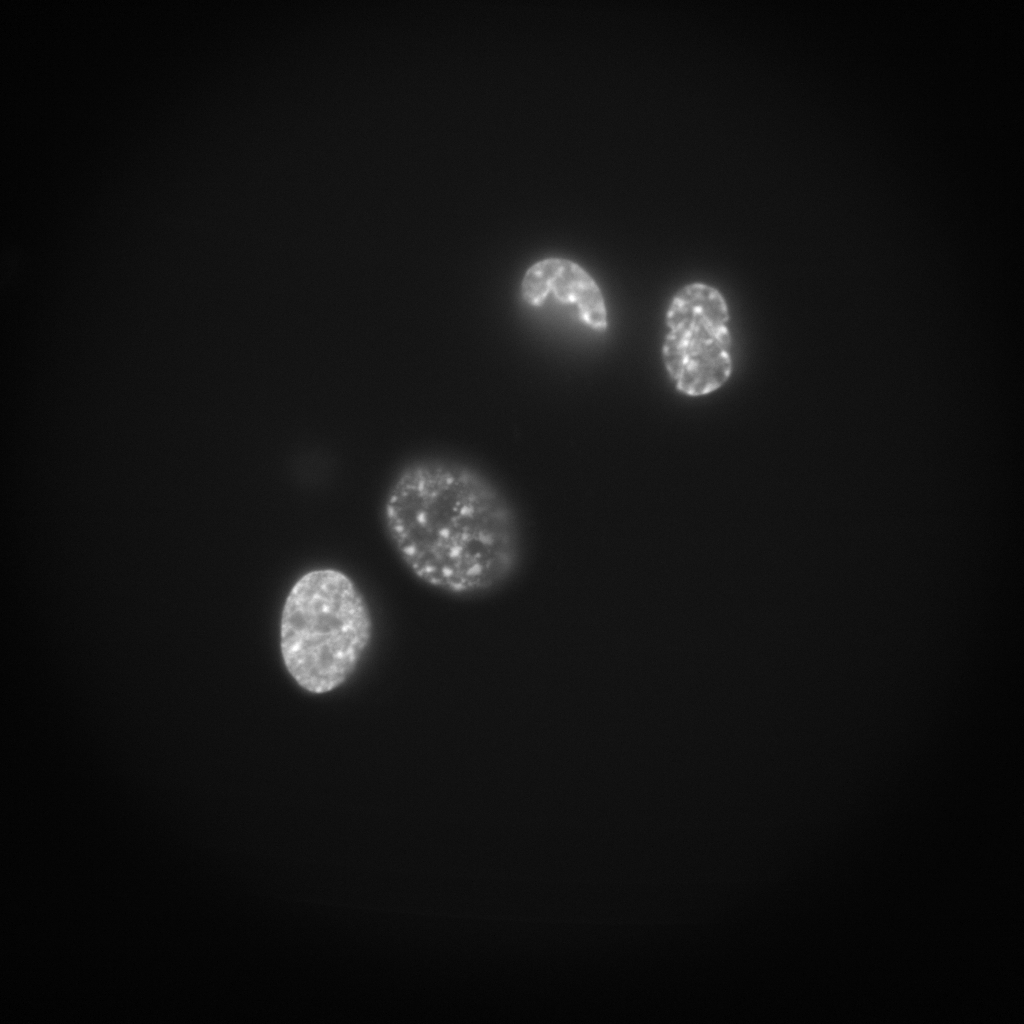

Supplement: Supplementary file 7 — Source Data [file 41467_2024_54141_MOESM7_ESM.zip › Fig 2c/LatB/Fig.2C LatB DAPI.tif]

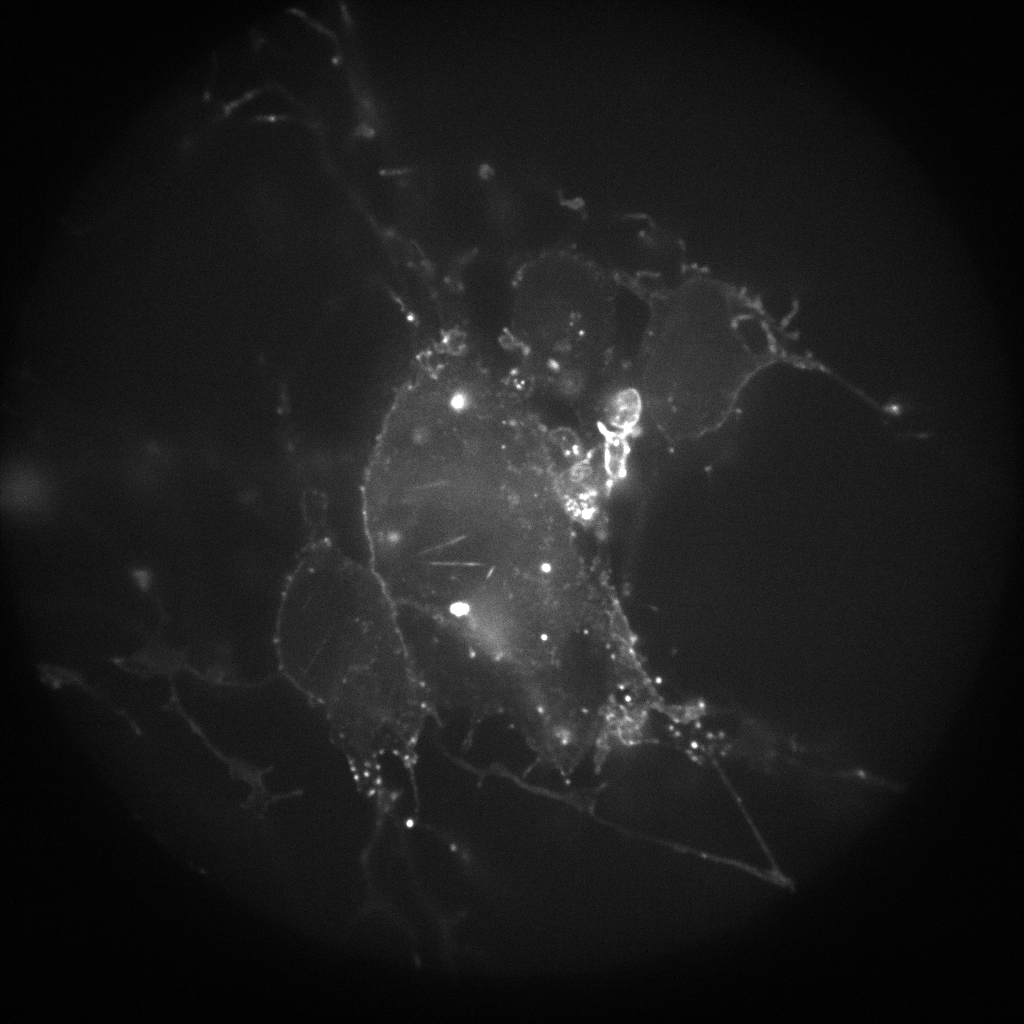

Supplement: Supplementary file 7 — Source Data [file 41467_2024_54141_MOESM7_ESM.zip › Fig 2c/LatB/Fig.2C LatB SiR-actin.tif]

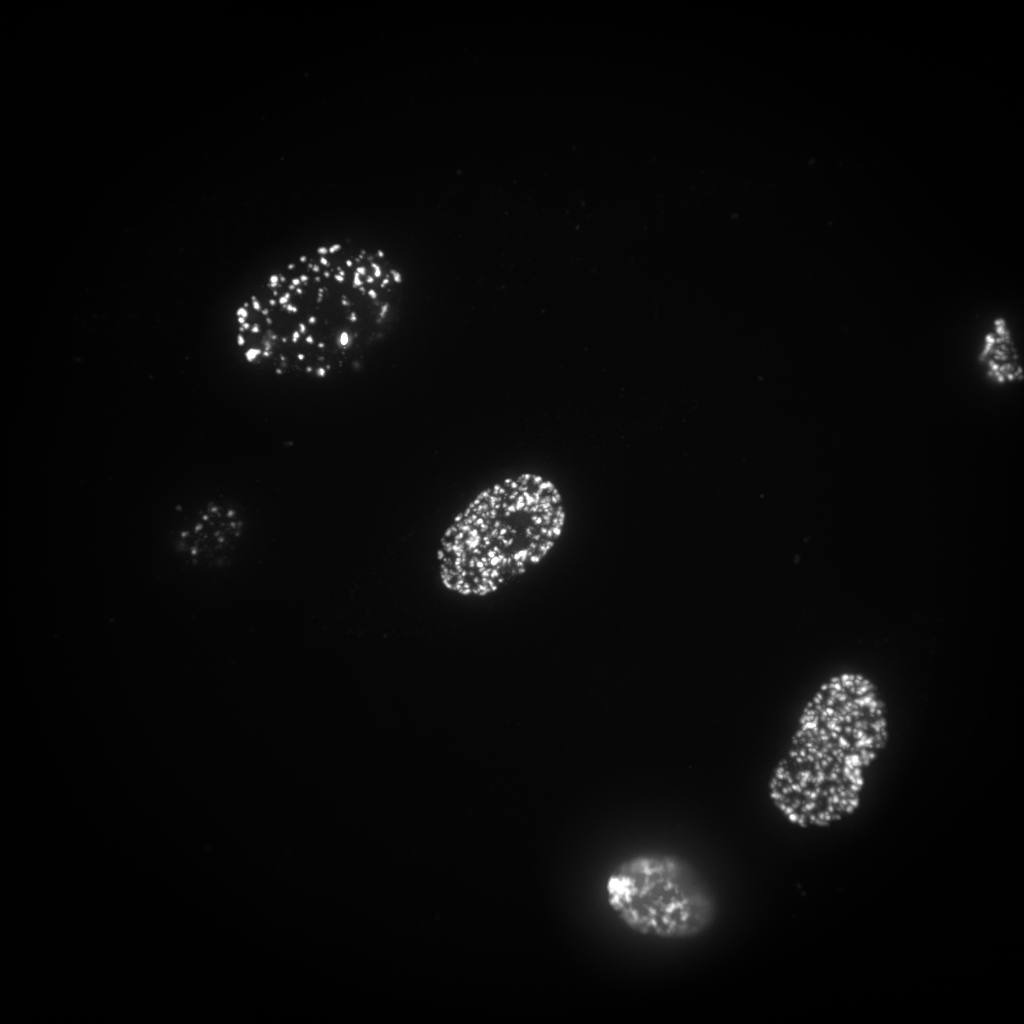

Supplement: Supplementary file 7 — Source Data [file 41467_2024_54141_MOESM7_ESM.zip › Fig 2c/LatB+Zeo/Fig.2C LatB+Zeo anti-phospho-Histone H2A.X.tif]

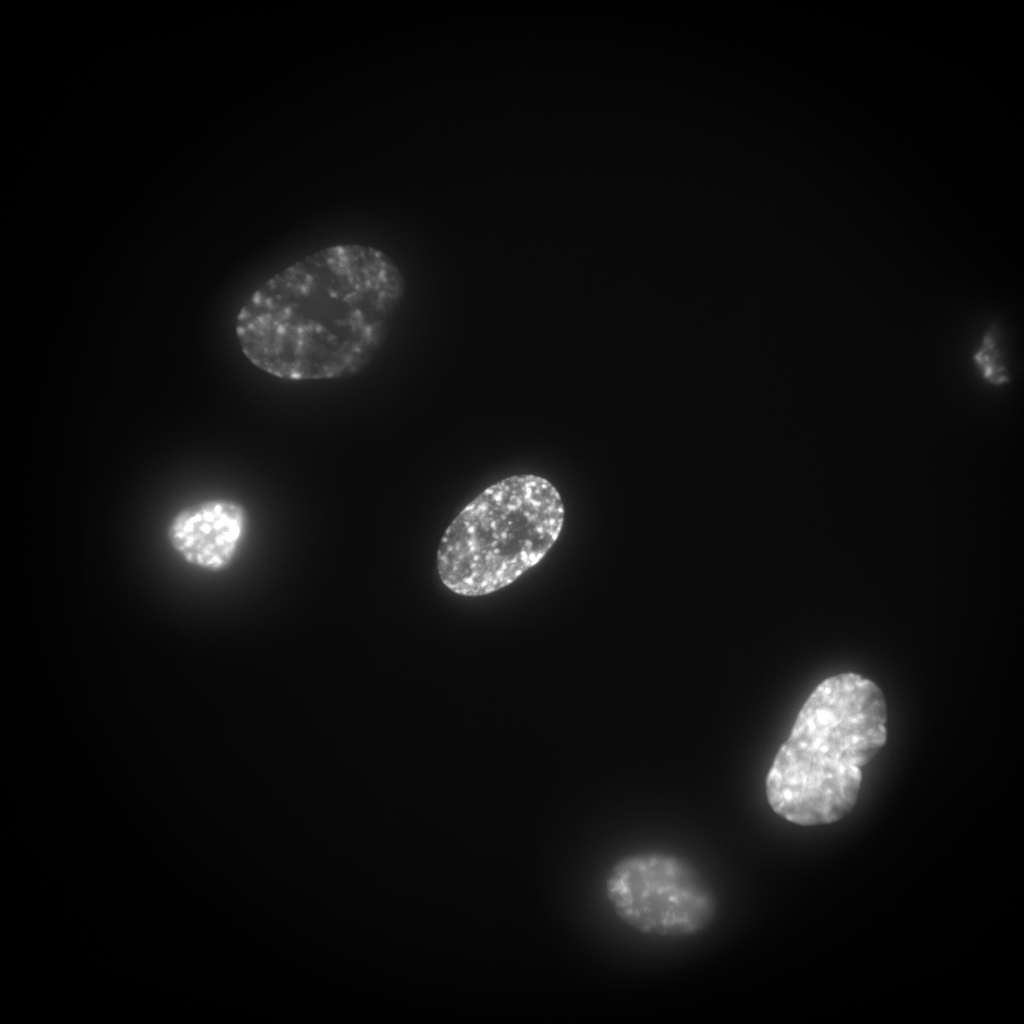

Supplement: Supplementary file 7 — Source Data [file 41467_2024_54141_MOESM7_ESM.zip › Fig 2c/LatB+Zeo/Fig.2C LatB+Zeo DAPI.tif]

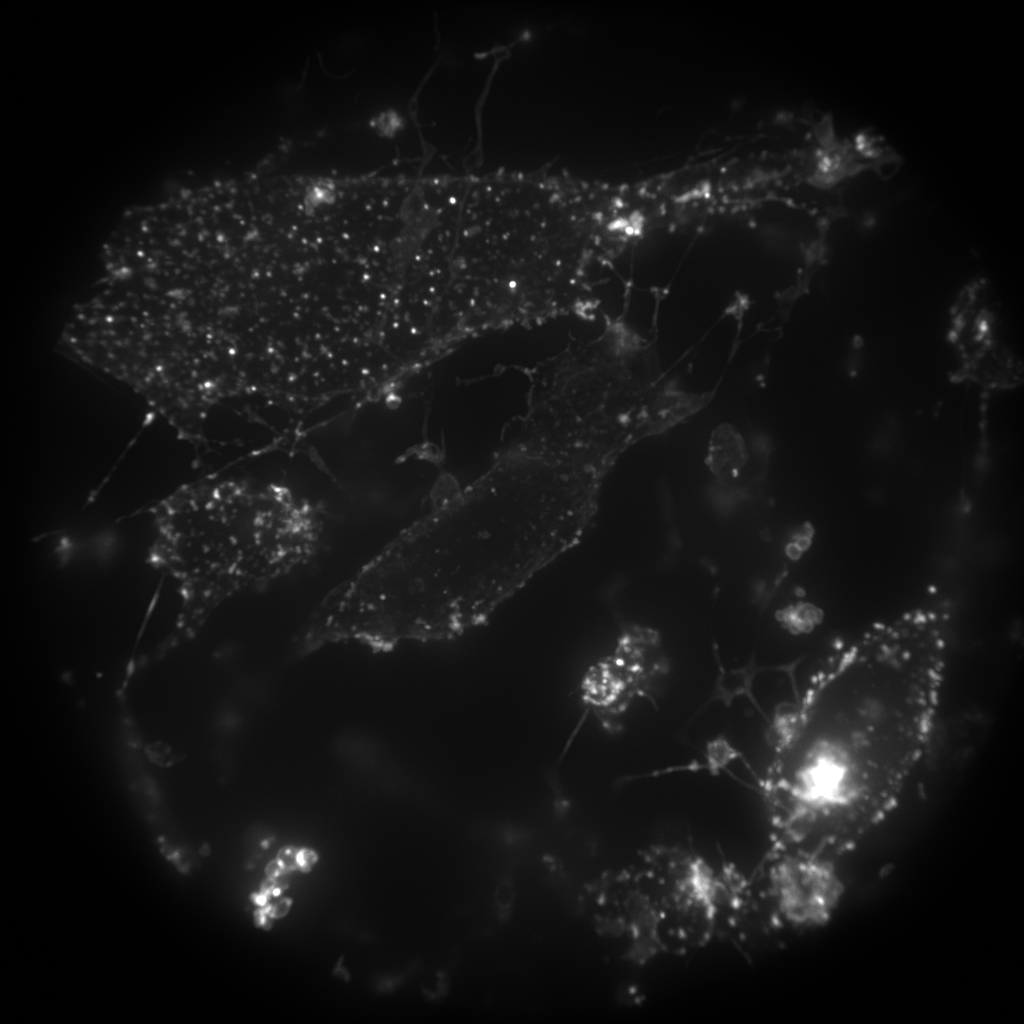

Supplement: Supplementary file 7 — Source Data [file 41467_2024_54141_MOESM7_ESM.zip › Fig 2c/LatB+Zeo/Fig.2C LatB+Zeo SiR-actin.tif]

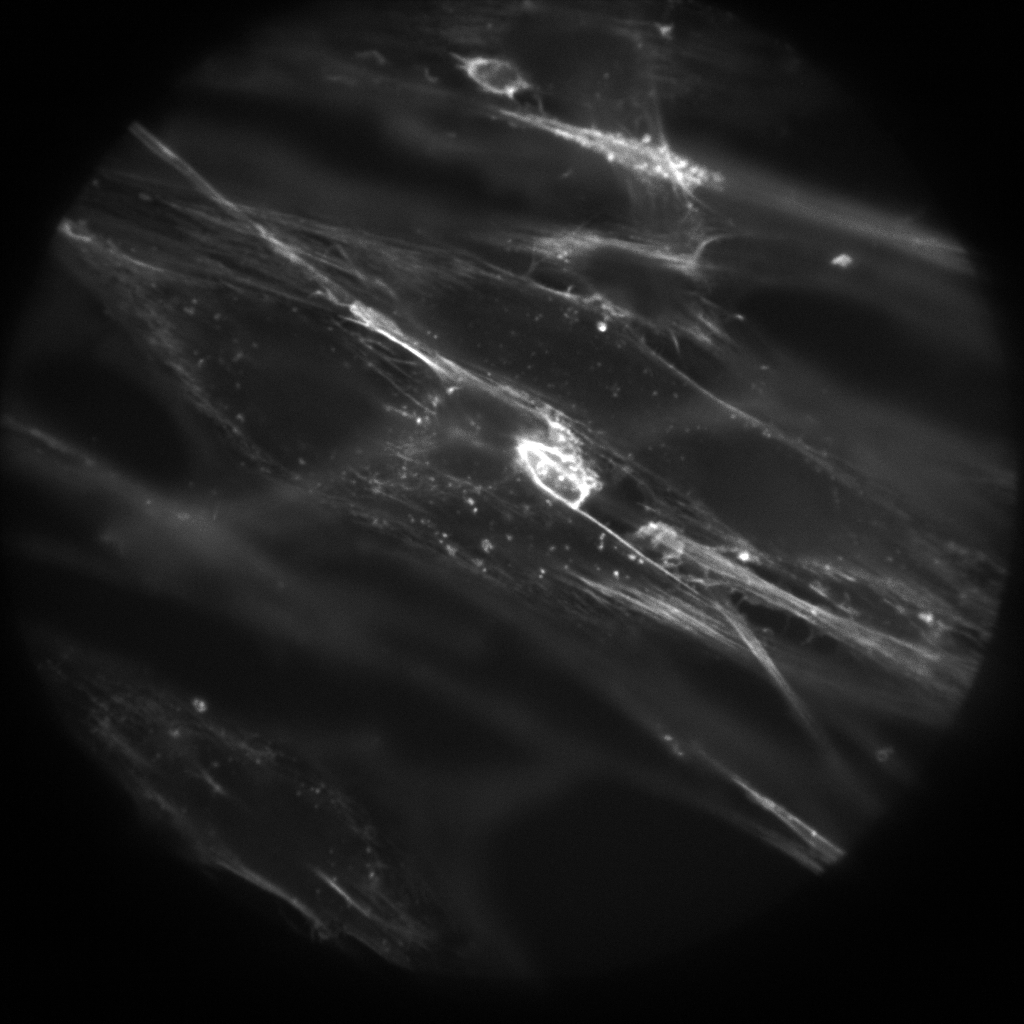

Supplement: Supplementary file 7 — Source Data [file 41467_2024_54141_MOESM7_ESM.zip › Fig 2c/Zeocin/Fig.2C Zeo anti-phospho-Histone H2A.X.tif]

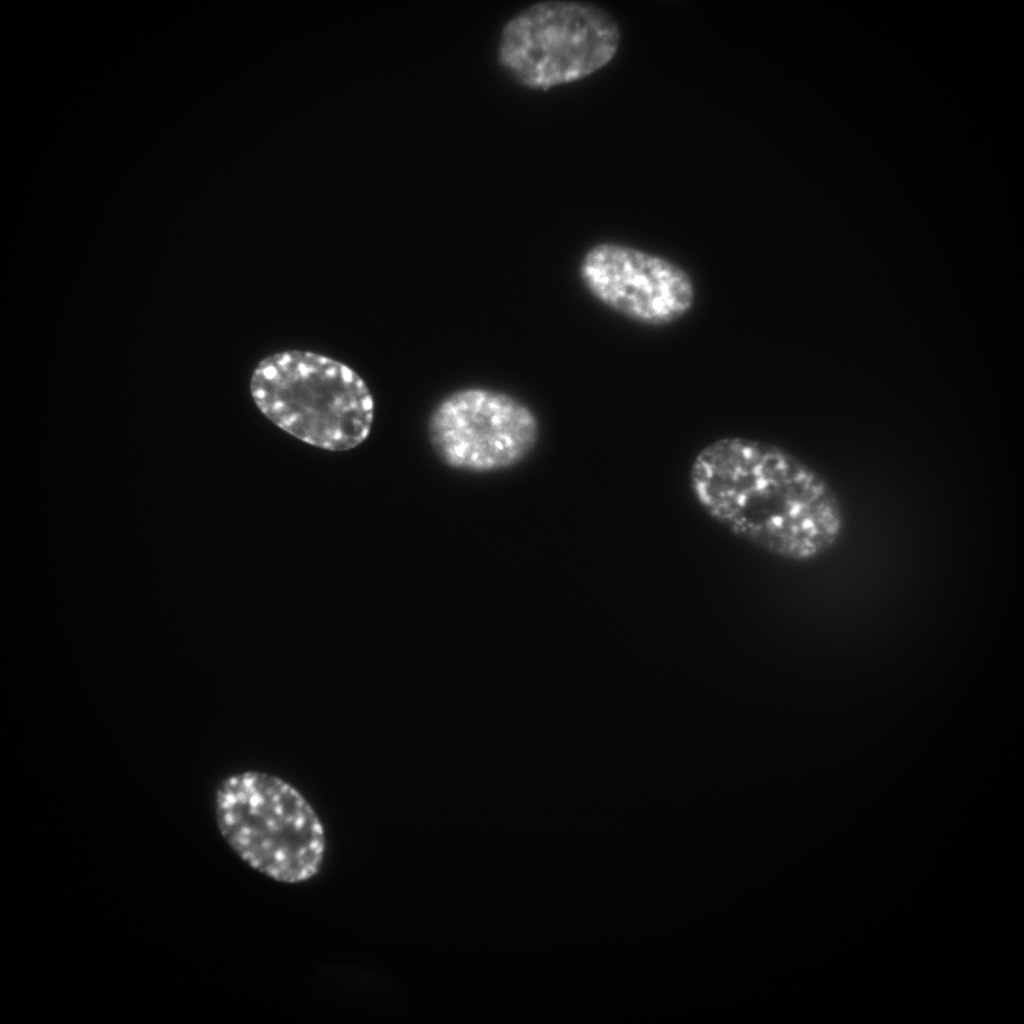

Supplement: Supplementary file 7 — Source Data [file 41467_2024_54141_MOESM7_ESM.zip › Fig 2c/Zeocin/Fig.2C Zeo DAPI.tif]

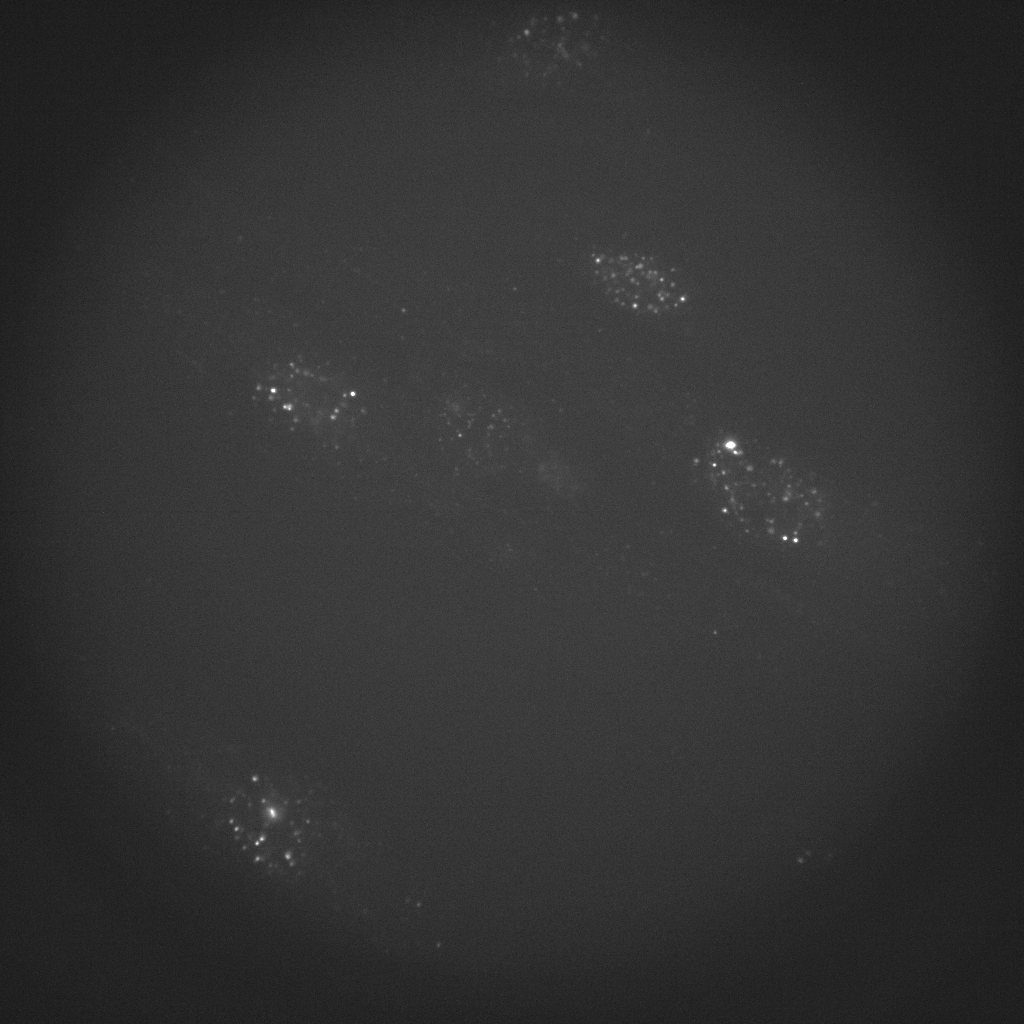

Supplement: Supplementary file 7 — Source Data [file 41467_2024_54141_MOESM7_ESM.zip › Fig 2c/Zeocin/Fig.2C Zeo SiR-actin.tif]

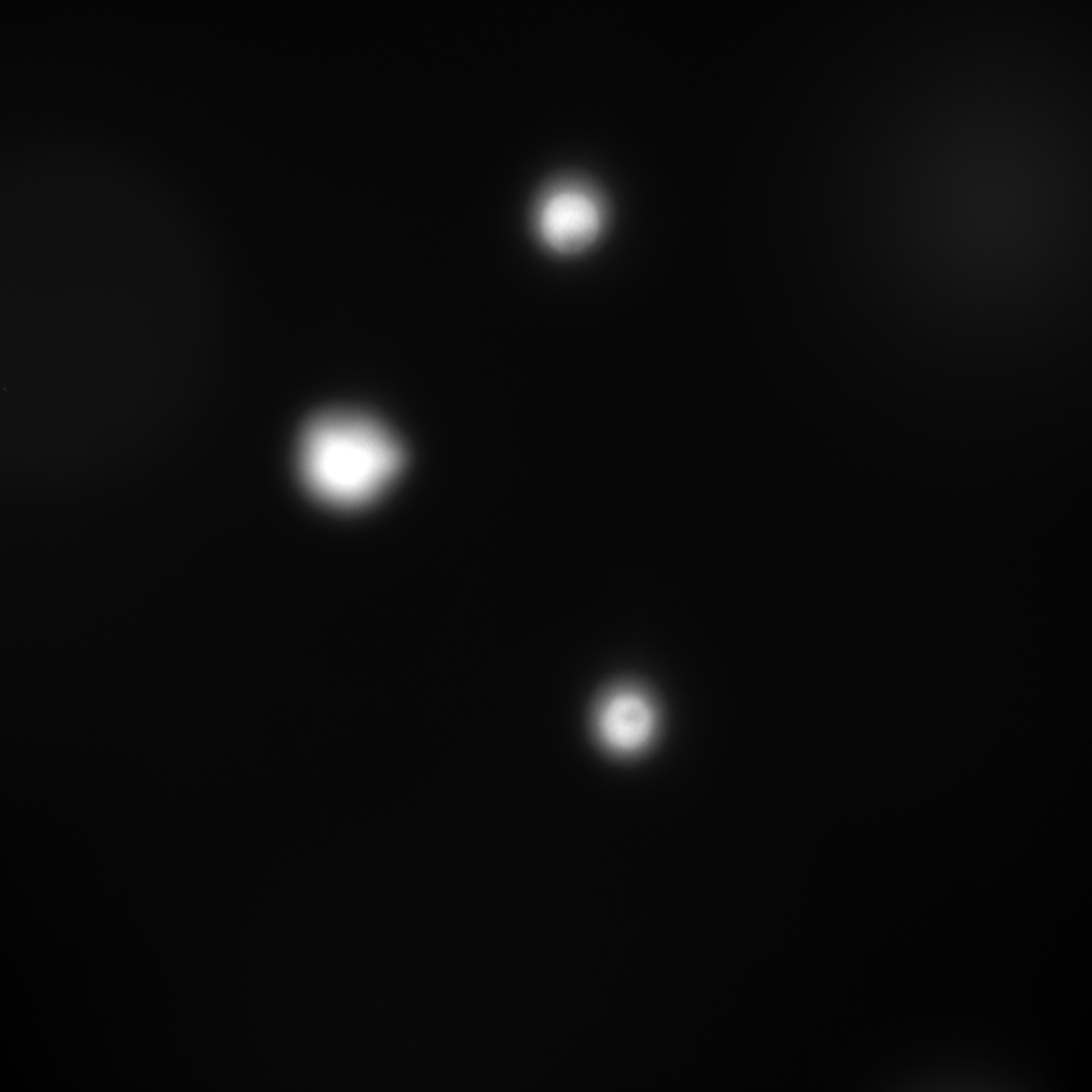

Supplement: Supplementary file 7 — Source Data [file 41467_2024_54141_MOESM7_ESM.zip › Fig 2d/Fig.2D DMSO.tif]

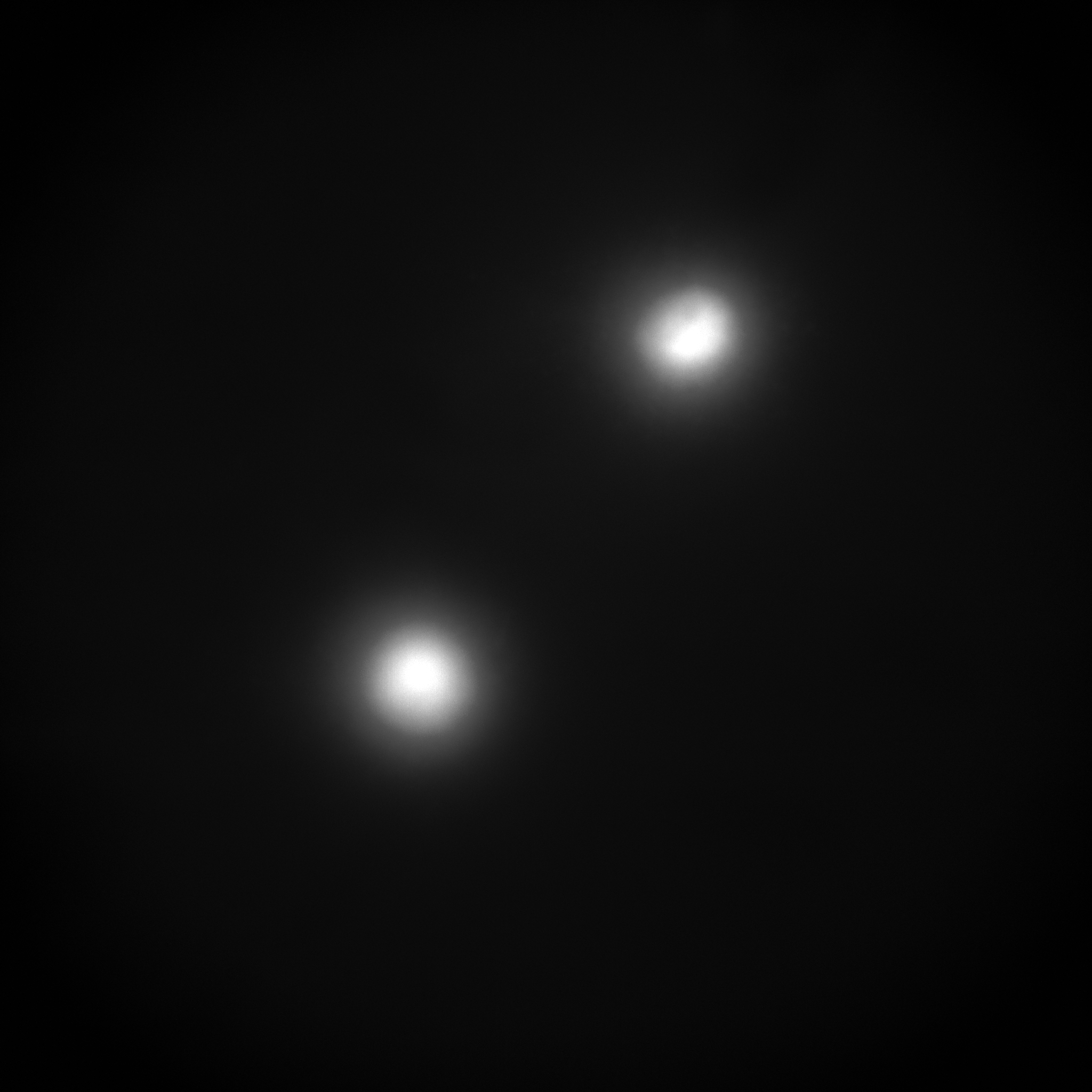

Supplement: Supplementary file 7 — Source Data [file 41467_2024_54141_MOESM7_ESM.zip › Fig 2d/Fig.2D LatB.tif]

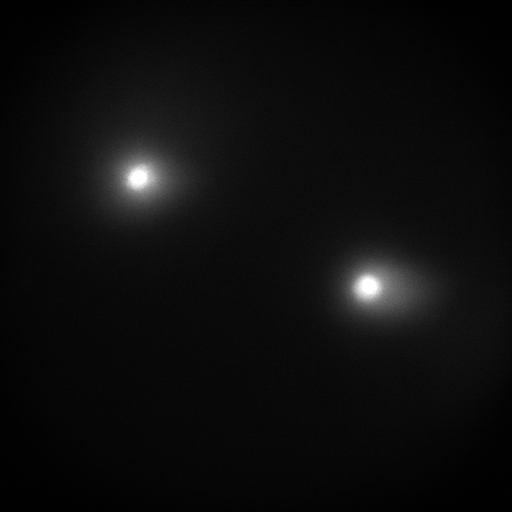

Supplement: Supplementary file 7 — Source Data [file 41467_2024_54141_MOESM7_ESM.zip › Fig 2d/Fig.2D Zeocin+LatB.tif]

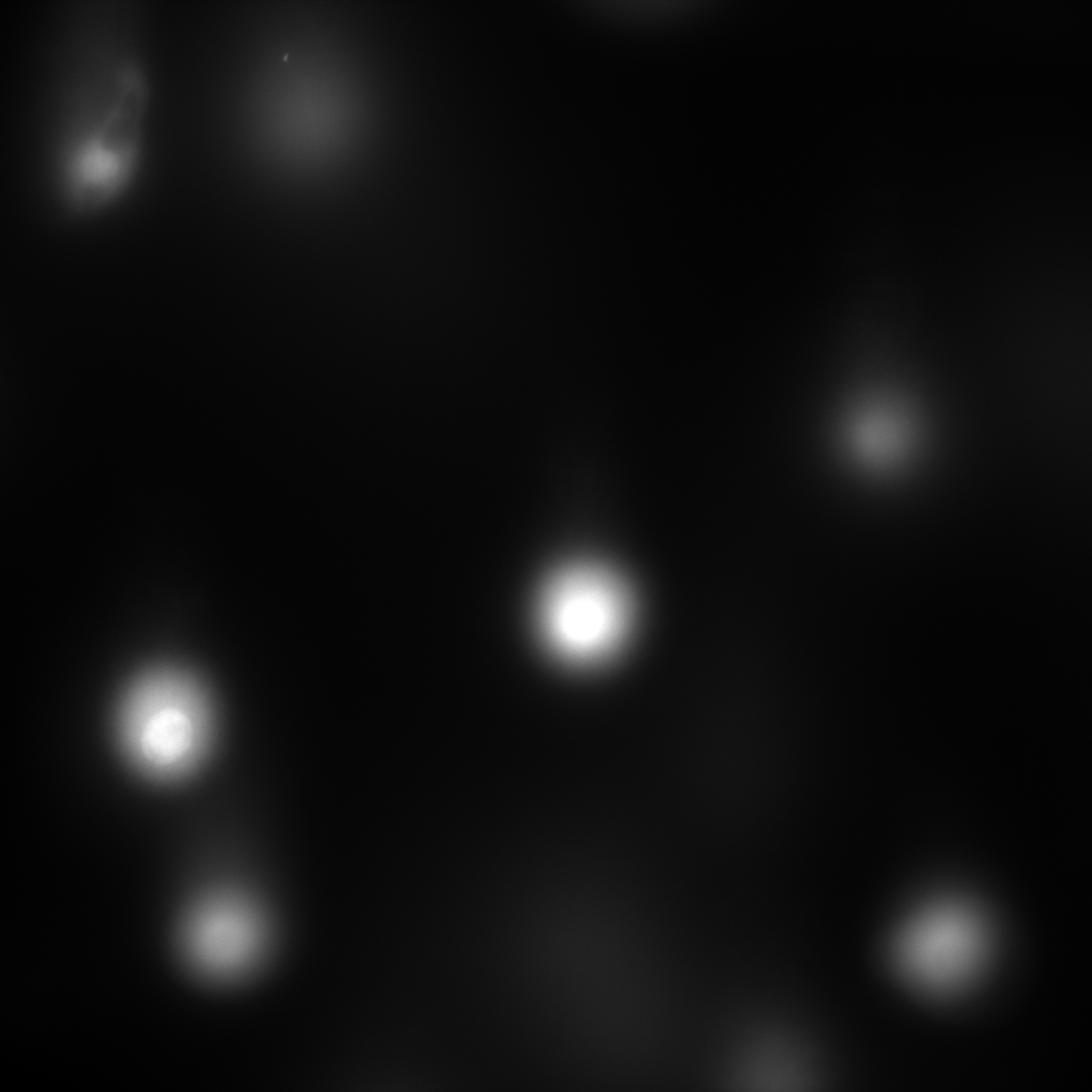

Supplement: Supplementary file 7 — Source Data [file 41467_2024_54141_MOESM7_ESM.zip › Fig 2d/Fig.2D Zeocin-adj.tif]

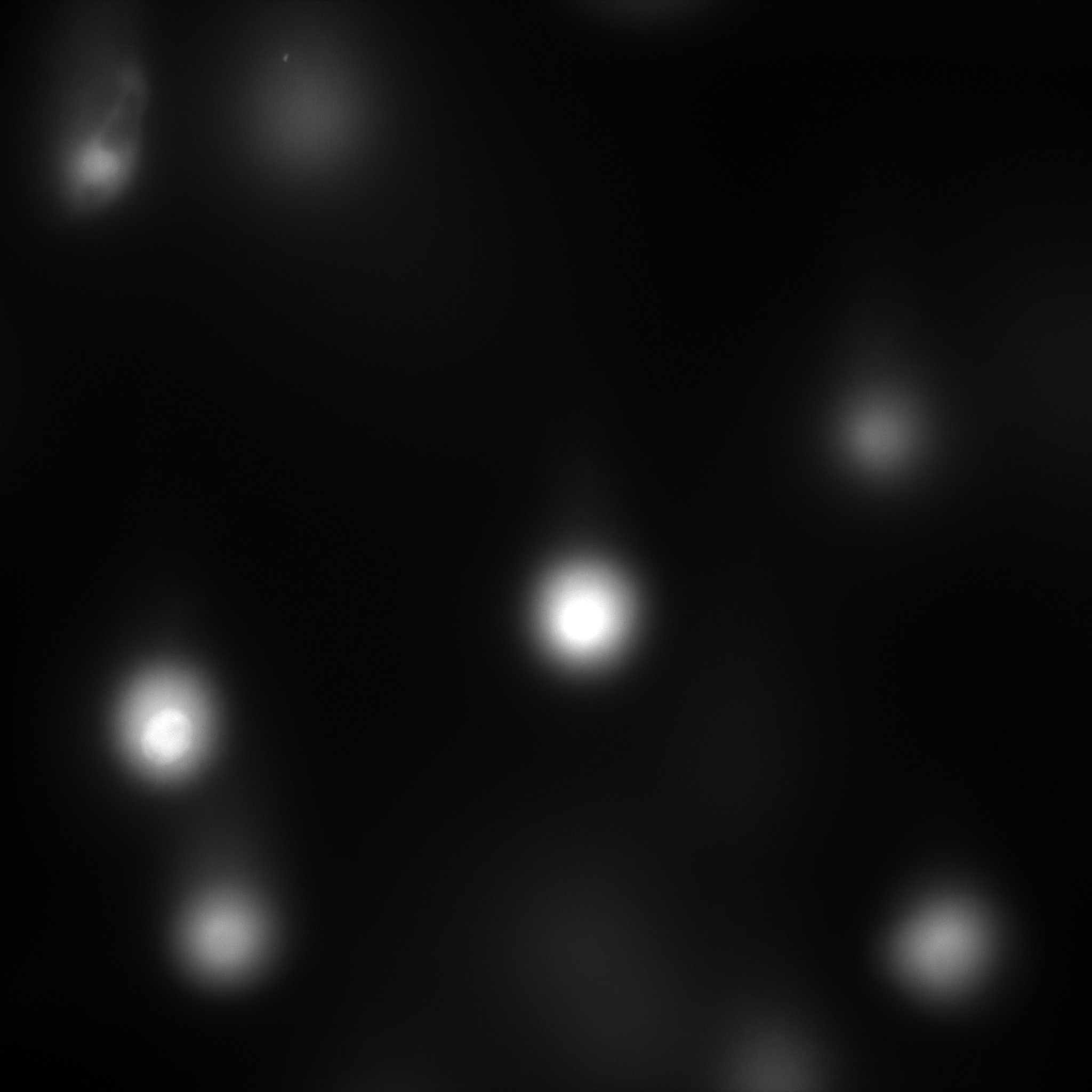

Supplement: Supplementary file 7 — Source Data [file 41467_2024_54141_MOESM7_ESM.zip › Fig 2d/Fig.2D Zeocin.tif]

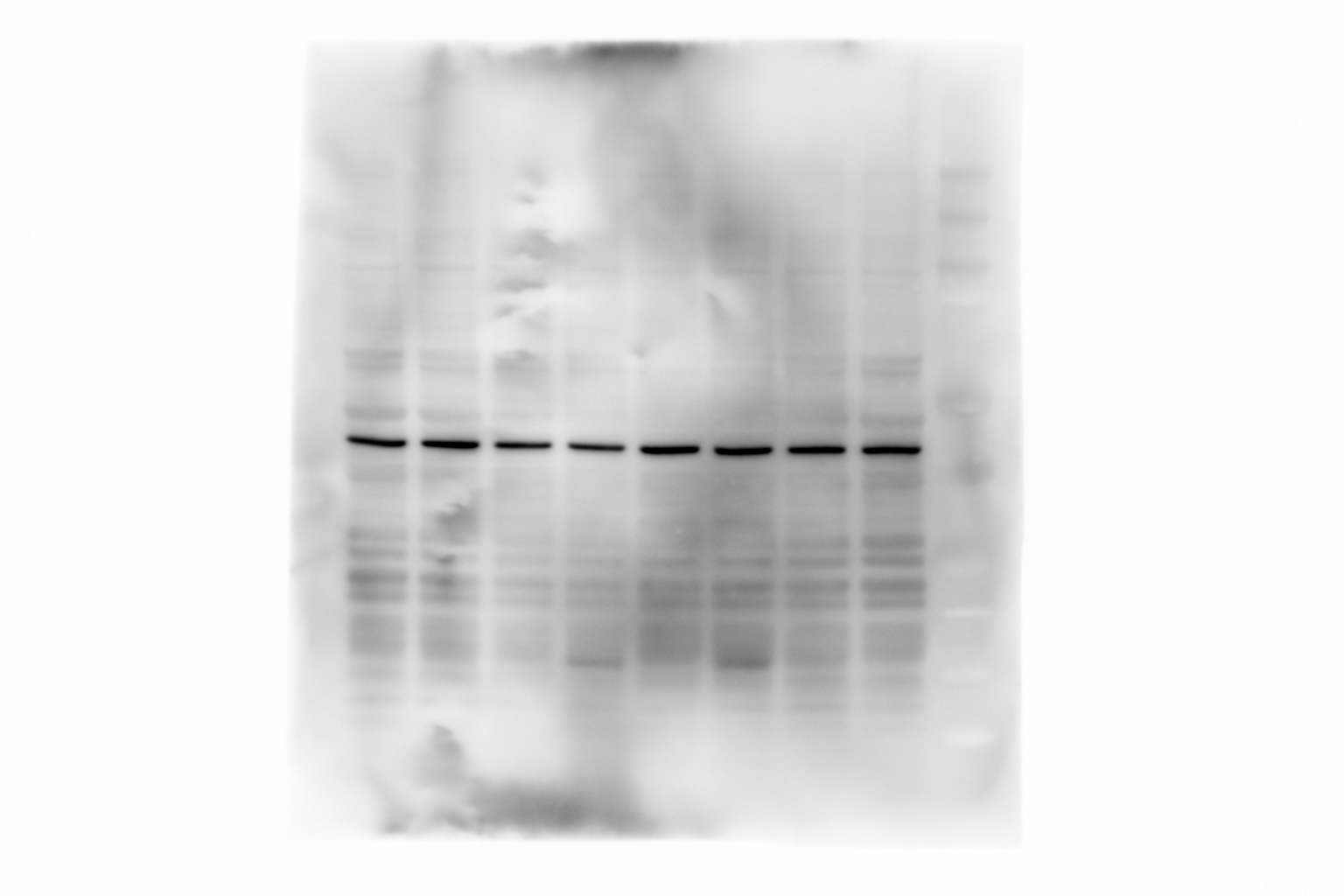

Supplement: Supplementary file 7 — Source Data [file 41467_2024_54141_MOESM7_ESM.zip › Hurst et al_uncropped blots/Fig1C/20160629_anti-actin.tif]

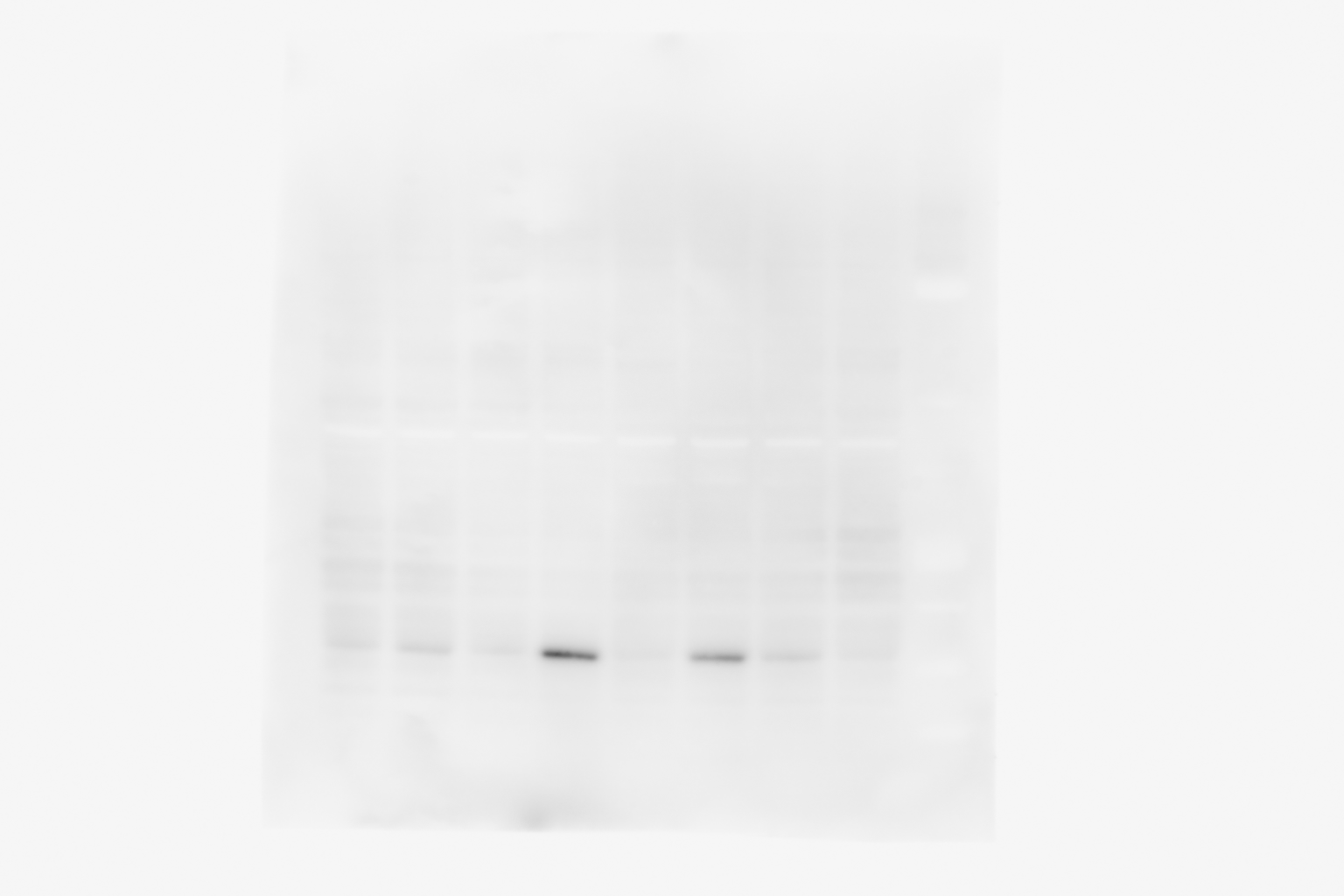

Supplement: Supplementary file 7 — Source Data [file 41467_2024_54141_MOESM7_ESM.zip › Hurst et al_uncropped blots/Fig1C/2016_06_27_H2A-p.tif]

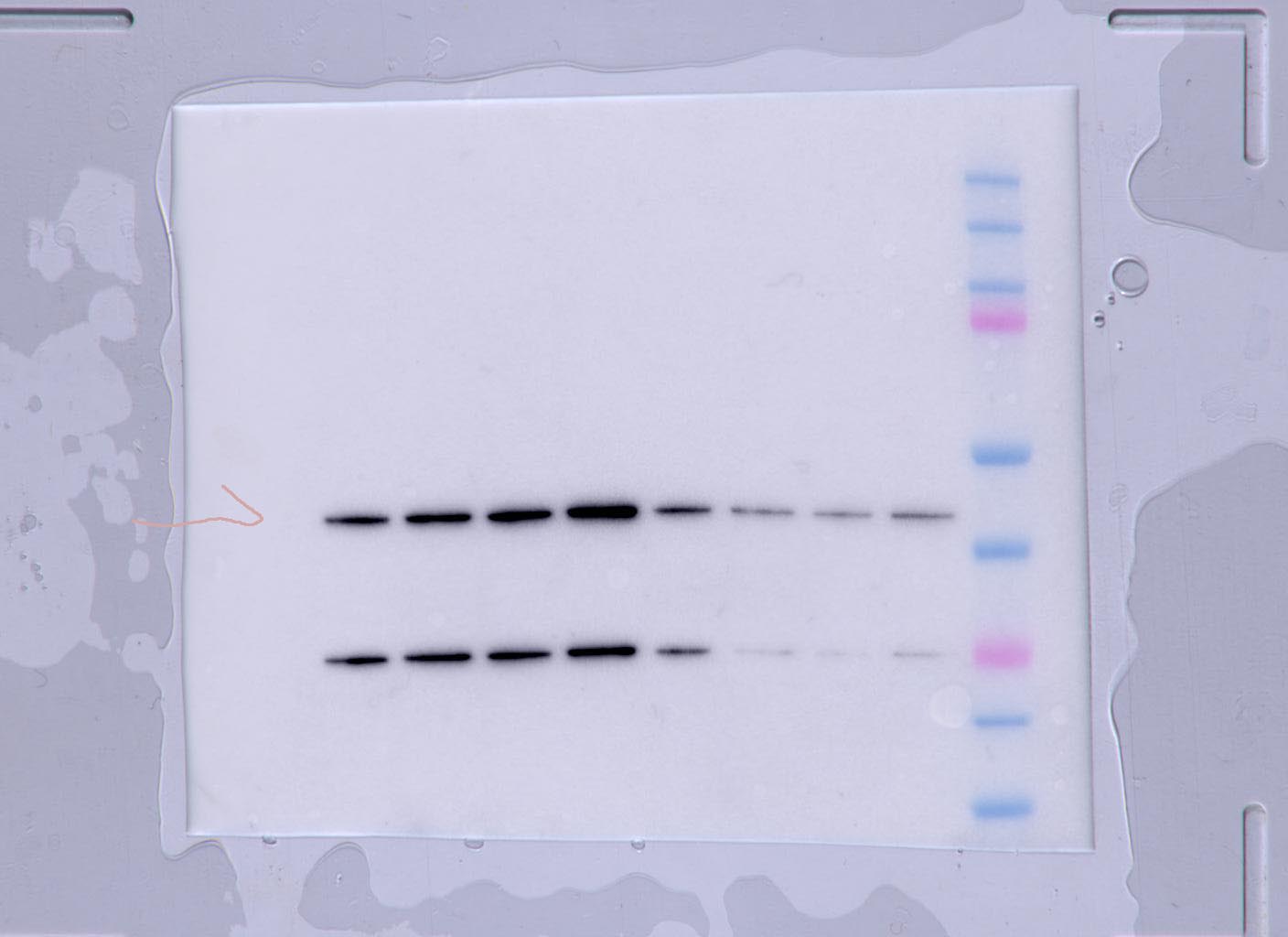

Supplement: Supplementary file 7 — Source Data [file 41467_2024_54141_MOESM7_ESM.zip › Hurst et al_uncropped blots/Fig6D/6D_bottom/a-act_set2+Marker.jpg]

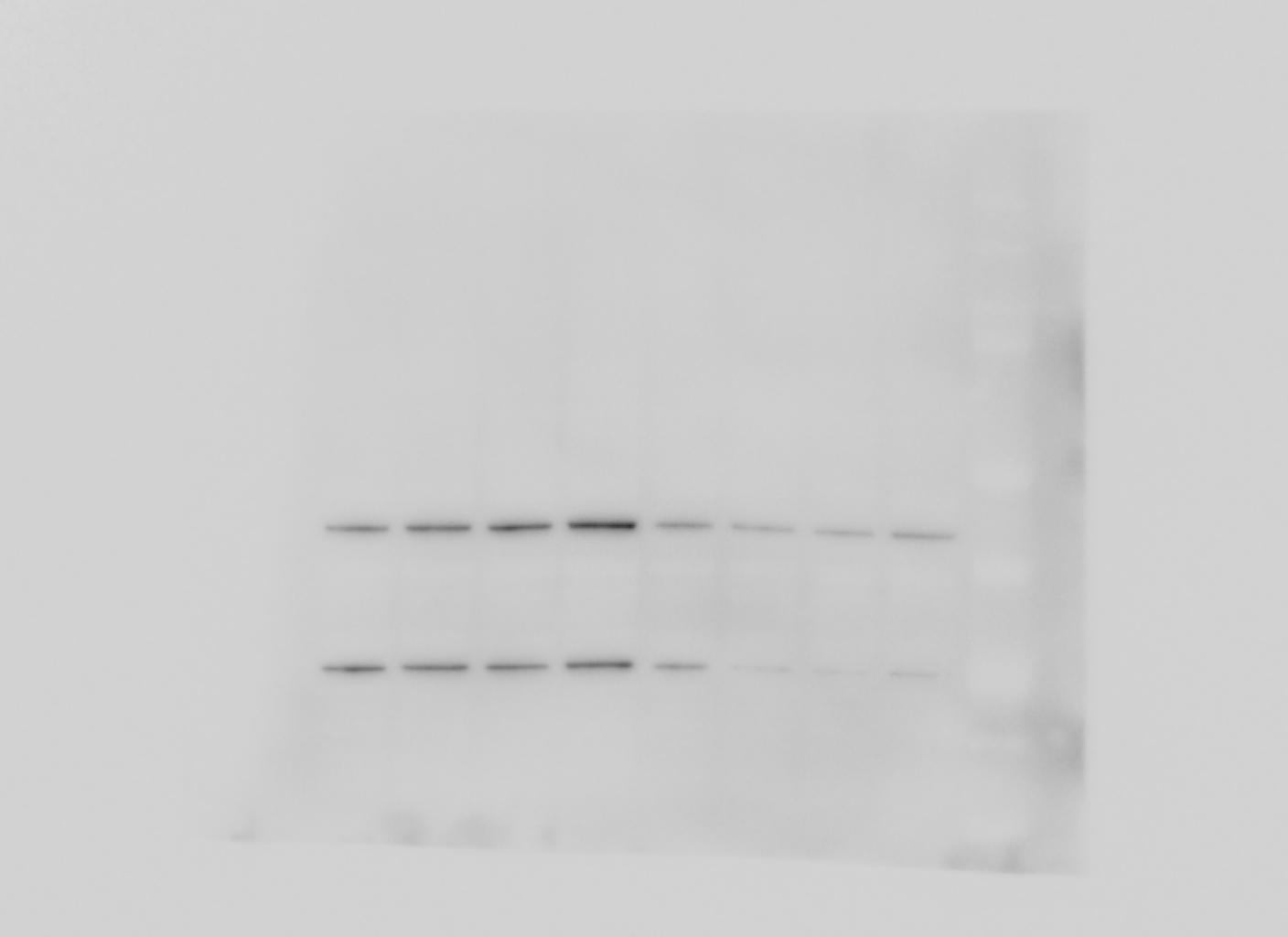

Supplement: Supplementary file 7 — Source Data [file 41467_2024_54141_MOESM7_ESM.zip › Hurst et al_uncropped blots/Fig6D/6D_bottom/a-act_set2_20s_used in the paper.tif]

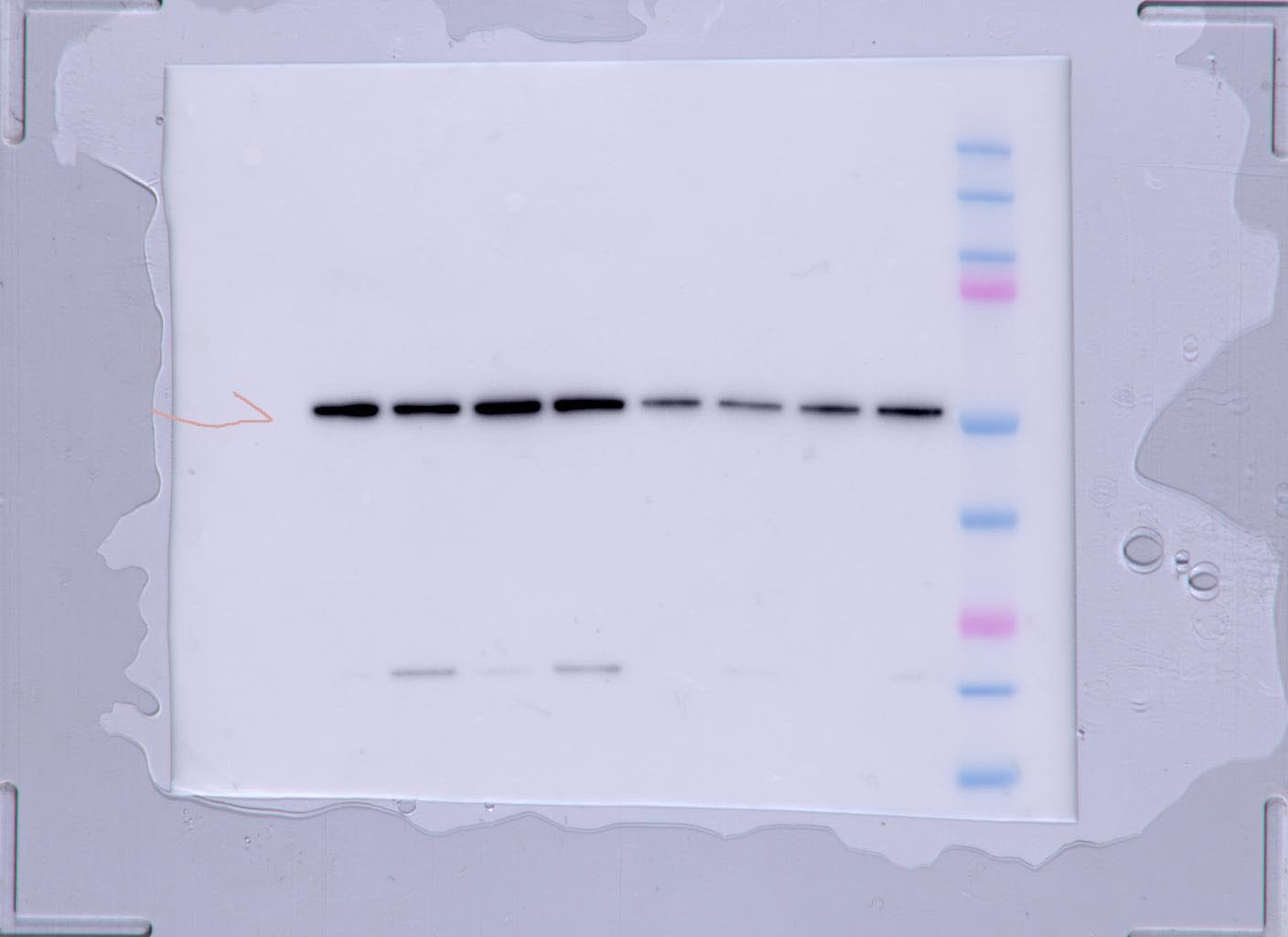

Supplement: Supplementary file 7 — Source Data [file 41467_2024_54141_MOESM7_ESM.zip › Hurst et al_uncropped blots/Fig6D/6D_bottom/a-tub_set2+Marker.jpg]

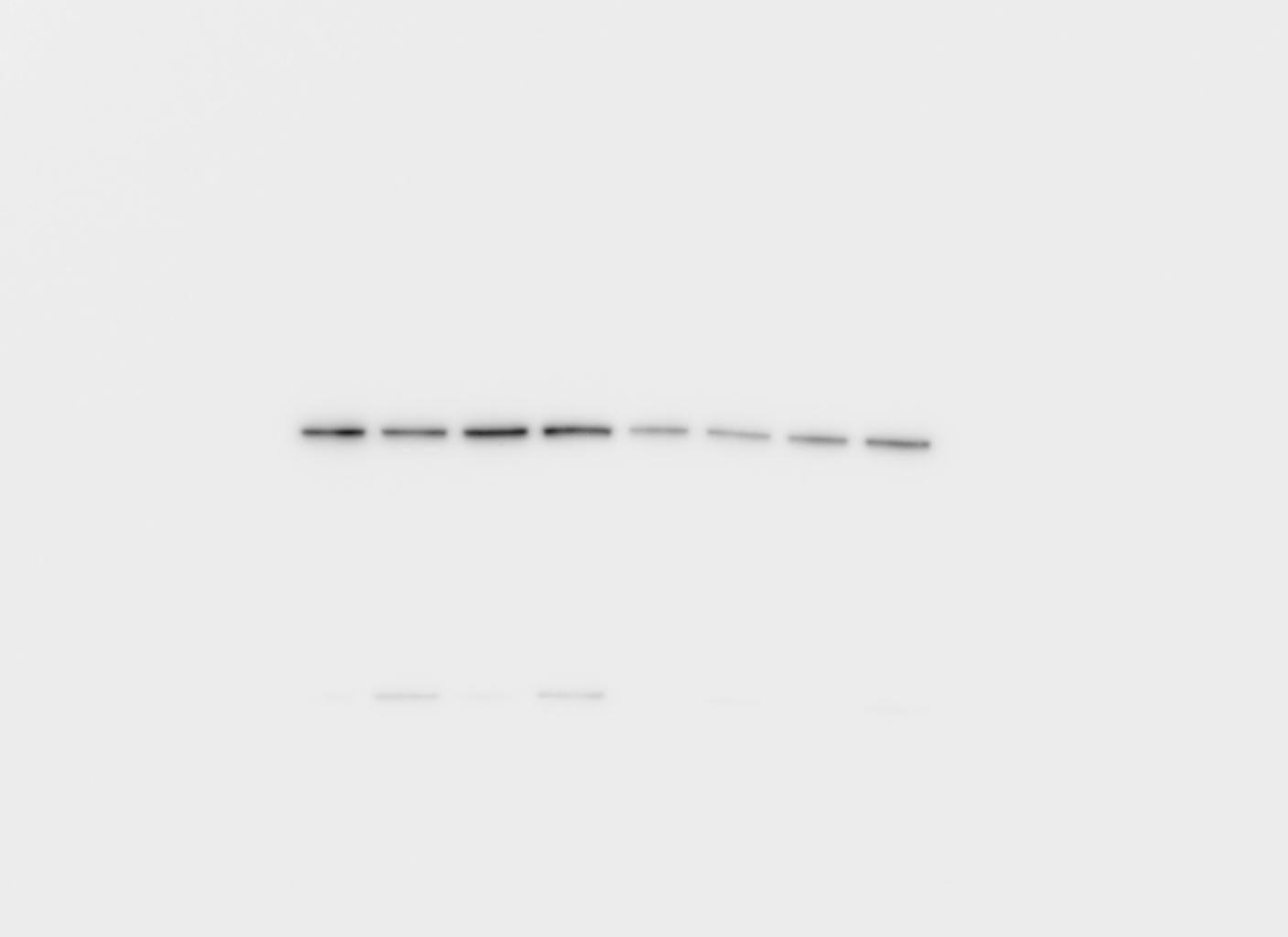

Supplement: Supplementary file 7 — Source Data [file 41467_2024_54141_MOESM7_ESM.zip › Hurst et al_uncropped blots/Fig6D/6D_bottom/a-tub_set2_20s_used in the paper.tif]

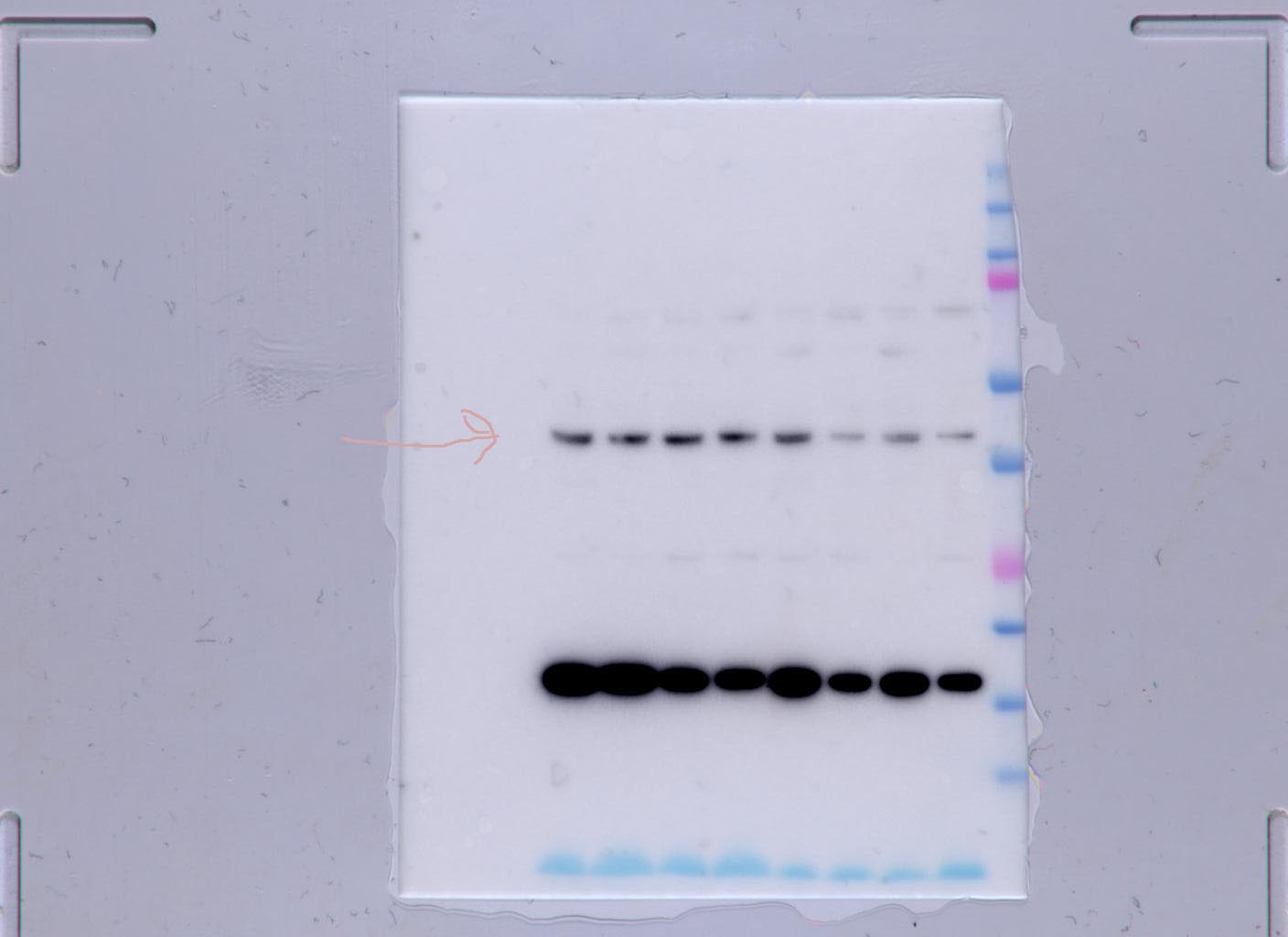

Supplement: Supplementary file 7 — Source Data [file 41467_2024_54141_MOESM7_ESM.zip › Hurst et al_uncropped blots/Fig7C/anti-ACTA1-set1+Marker.jpg]

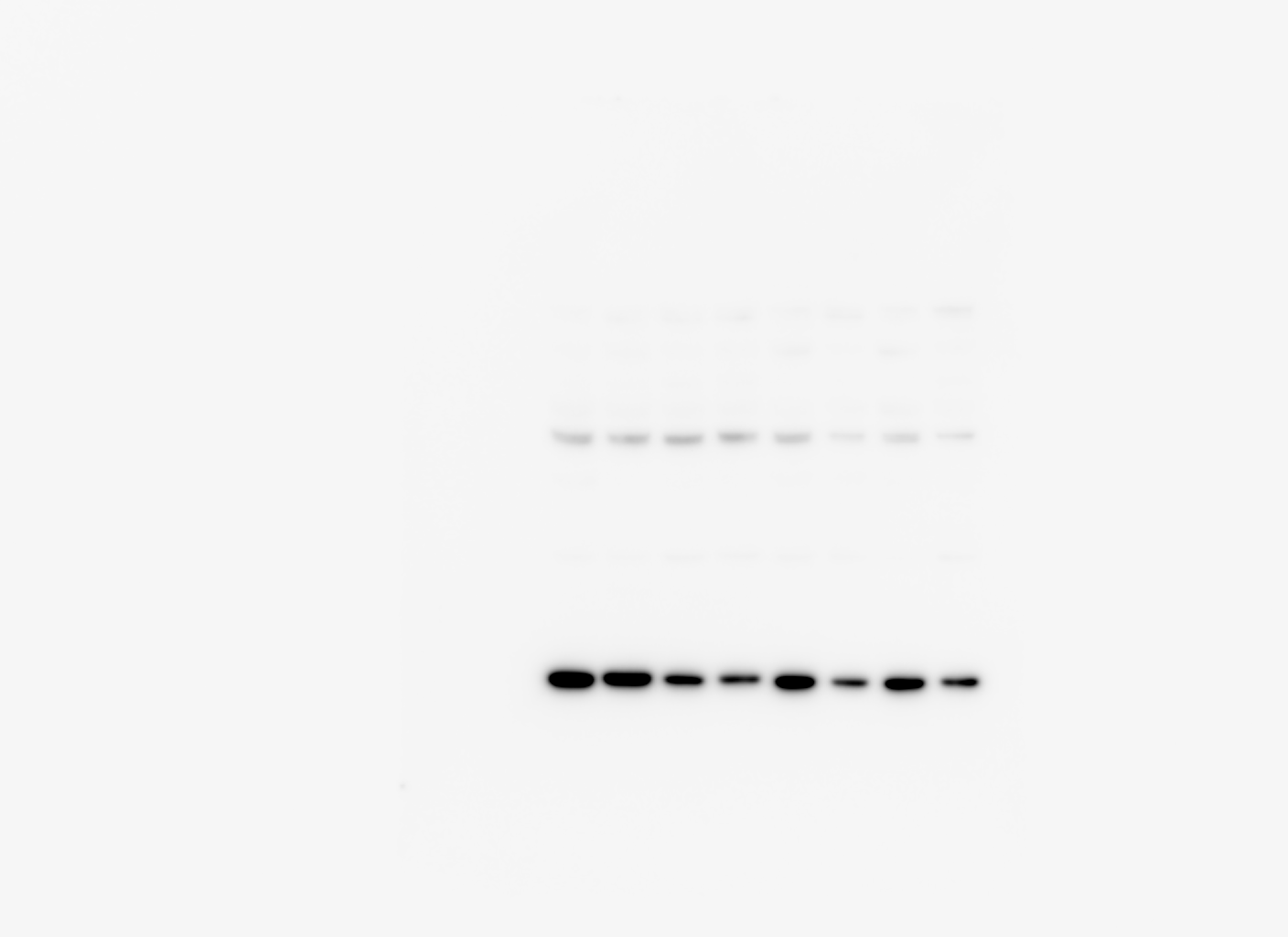

Supplement: Supplementary file 7 — Source Data [file 41467_2024_54141_MOESM7_ESM.zip › Hurst et al_uncropped blots/Fig7C/anti-ACTA1-set1.tif]

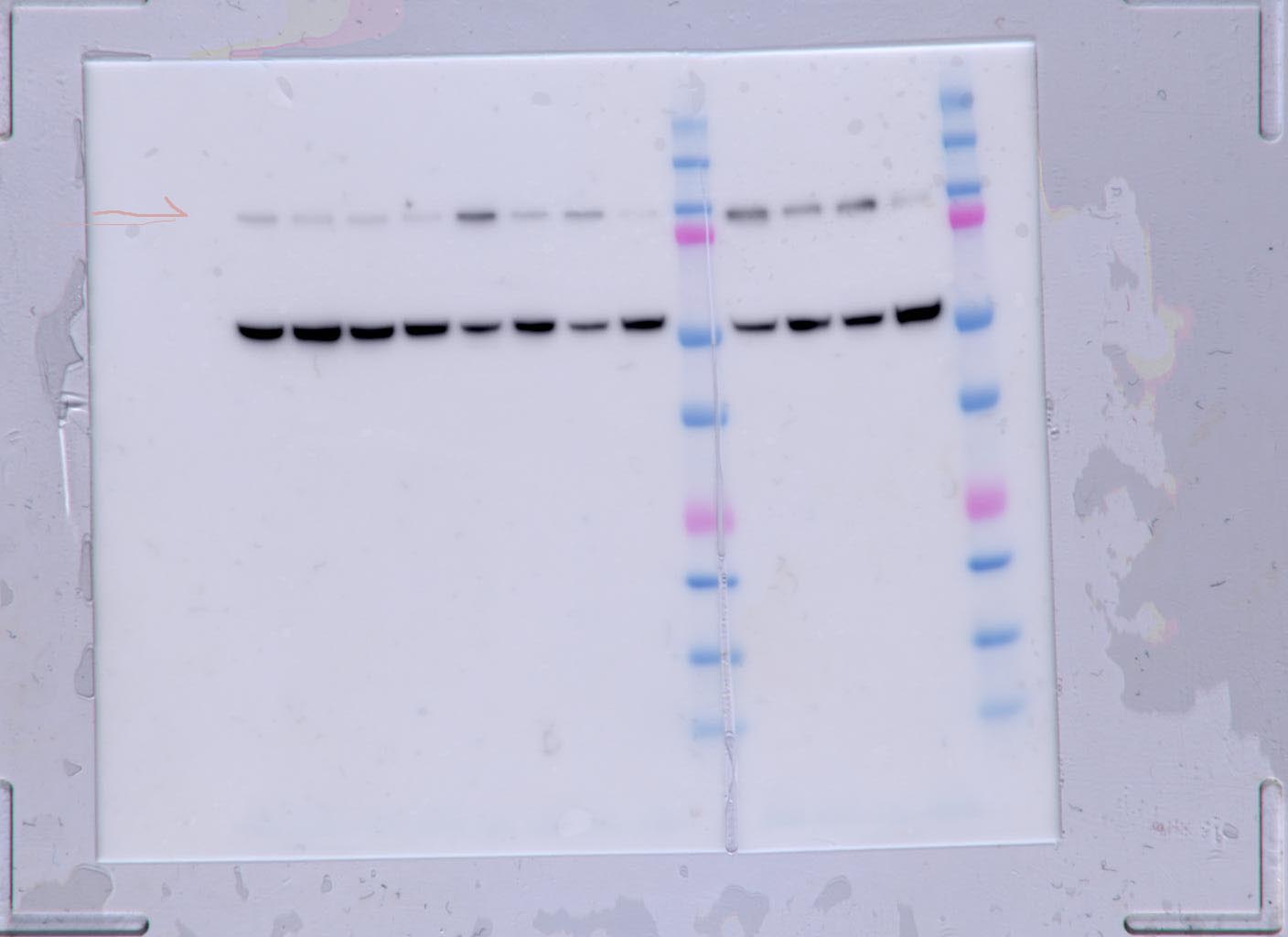

Supplement: Supplementary file 7 — Source Data [file 41467_2024_54141_MOESM7_ESM.zip › Hurst et al_uncropped blots/Fig7C/m414_TCE_set1+2+Marker.jpg]

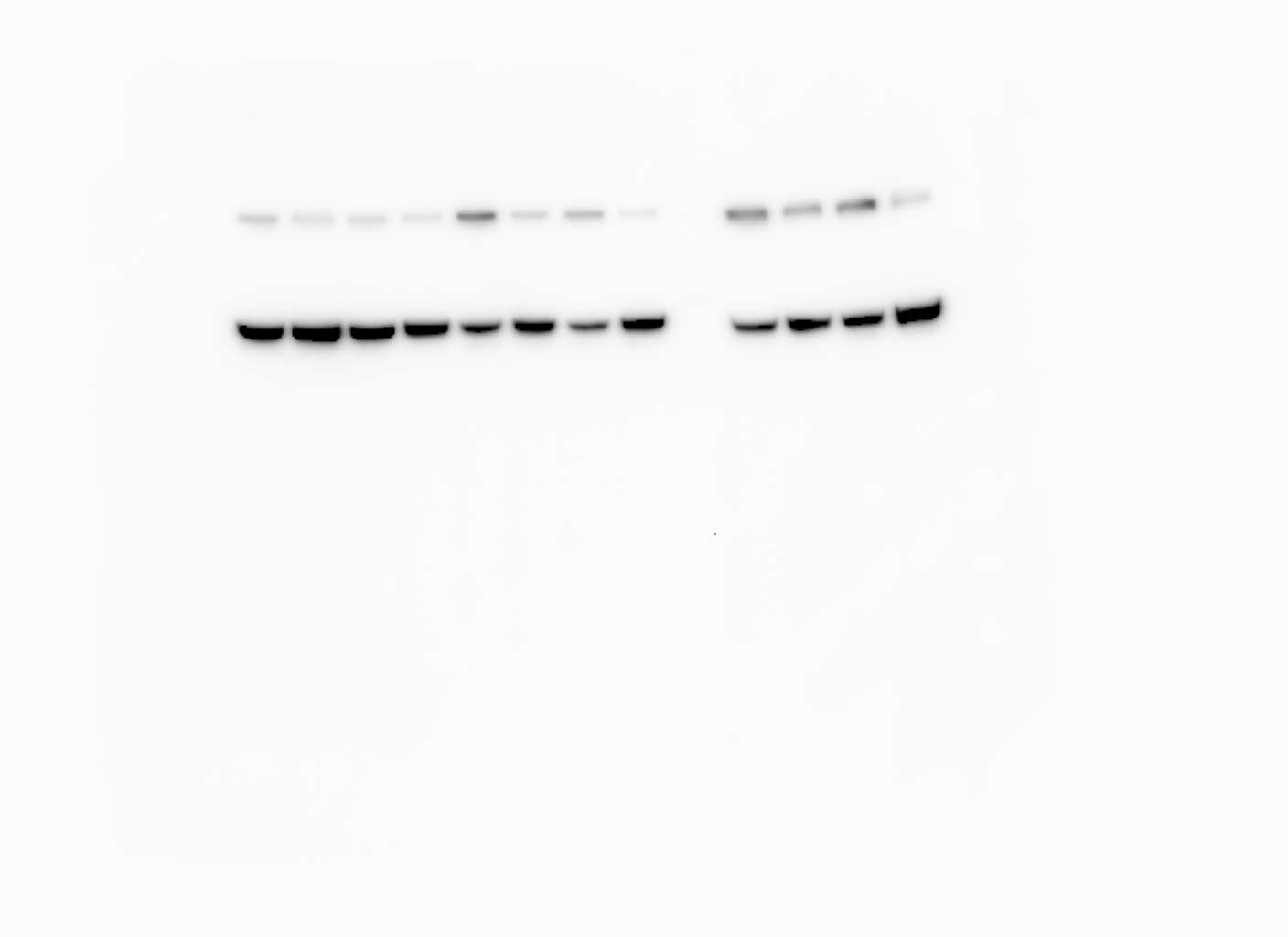

Supplement: Supplementary file 7 — Source Data [file 41467_2024_54141_MOESM7_ESM.zip › Hurst et al_uncropped blots/Fig7C/m414_TCE_set1+2.tif]
